# Implementation and lessons learned from 2 online interprofessional faculty development programs for improving educational practice in the health professions in Chile and the United Kingdom from 2018 to 2021

**DOI:** 10.3352/jeehp.2021.18.21

**Published:** 2021-08-09

**Authors:** Cesar Orsini, Veena Rodrigues, Jorge Tricio

**Affiliations:** 1 Norwich Medical School, Faculty of Medicine and Health Sciences, University of East Anglia, Norwich, UK; 2 Faculty Development Office, Faculty of Dentistry, Universidad de Los Andes, Santiago, Chile; Hallym University, Korea

**Keywords:** Distance education, Health occupations, Interdisciplinary studies, Interprofessional education, Problem-based learning

## Abstract

This study presents the design, implementation, and lessons learned from 2 fit-for-purpose online interprofessional faculty development programs for educational practice improvement in the health professions in Chile and the United Kingdom from 2018 to 2021. Both programs were designed to enhance teaching and learning practices in an interprofessional environment based on 4 pillars: professional diversity, egalitarianism, blended/online learning, and active learning strategies. A multidisciplinary mix of educators participated, showing similar results. The 3 main lessons learned were that the following factors facilitated an interprofessional environment: a professions-inclusive teaching style, a flexible learning climate, and interprofessional peer work. These lessons may be transferable to other programs seeking to enhance and support interprofessionality. Faculty development initiatives preparing educators for interprofessional practice should be an integral component of health professions education, as delivering these courses within professional silos is no longer justifiable. As the relevance of interprofessional education grows, an effective way of promoting interprofessonal education is to train the trainers in formal interprofessional settings.

## Introduction

### Background/rationale

Well-designed interprofessional education (IPE) programs among learners in the health professions have been found to foster professional practice, teamwork, communication, and valuing the role of others in the healthcare team [1,2]. On the contrary, poorly planned and delivered IPE initiatives may generate reluctance to engage in interprofessional collaboration and reinforce stereotypes [2,3]. Educator preparation has been reported as a critical factor supporting IPE success, especially considering the integration of IPE into accreditation standards across disciplines [4]. Therefore, to foster an optimal IPE environment, educators themselves need to support IPE and ideally to have experienced IPE in order to model the best educational methods and practices [5]. This points to the need for advanced educational training, as the design and facilitation of interprofessional learning activities require significant educator preparation to ensure the correct level and allow all professional groups to contribute and learn with, from, and about each other. Therefore, educators must be prepared and trained to meet this challenge since they play a fundamental role in the teaching and learning of IPE.

Unfortunately, most health professions faculty development programs oriented towards improving educational practices, covering aspects such as instructional design, teaching and learning effectiveness, and evaluation strategies, have been traditionally delivered within uniprofessional or multiprofessional settings, with no evidence-based justification [6]. The concept of multiprofessional education (MPE), where members of different professions learn side by side without necessarily interacting, is often mistaken for and used interchangeably with IPE [2]. While MPE is frequently used when different professional groups have a common need to address, the distinctiveness of IPE lies in intentionally bringing together participants from different professions to focus on a particular topic or task with deliberate interactions. By doing so, IPE is expected to increase the diversity of knowledge and perspectives, enhancing the learning of all [1]. One way to support IPE is for educators from different disciplines to be trained in educational theory and practice in interprofessional settings to understand and become aware of each other’s disciplines, their teaching practices, and potential transferability.

An additional barrier for faculty development programs is the mode of delivery, as the majority are described as face-to-face, limiting attendance and engagement for busy clinical educators [6]. This is especially relevant considering the impact of the coronavirus disease 2019 (COVID-19) pandemic on healthcare educators and the transition to online learning [7]. One way to address these challenges and support IPE is to deliberately design online faculty development programs for clinical education with participants from different health disciplines actively learning with, from, and about each other’s professions and teaching methods.

### Objectives

Thus, the aim of this paper was to present the design, implementation, and lessons learned from 2 fit-for-purpose online IPE faculty development programs for educational practice improvement in the health professions.

### Ethics statement

This was not a human-subject study; therefore, neither receiving approval from the institutional review board nor obtaining informed consent was required.

### Study design

This study presents a narrative review of curriculum development.

## Design and implementation

The clinical education program (master’s, postgraduate diploma, and postgraduate certificate awards) at the University of East Anglia (UK) and the postgraduate diploma in health professions education at the University of the Andes (Chile) were designed to enhance teaching, learning, and assessment in an interprofessional learning environment. Both programs were designed to provide educators with the opportunity to understand the work ethos and professional values of colleagues from other health professions, along with experiencing interprofessional learning and collaborative work. Despite differences in geographical location, language, and culture, the courses exhibit numerous similarities. Table 1 illustrates the main features of both programs. In addition, both courses’ programs/handbooks can be found in Supplements 1 and 2.

The interprofessional environment was built on 4 pillars: professional diversity, egalitarianism, blended learning, and active learning strategies. The learning experience was enhanced by the diversity of core academic teams contributing to teaching and by educators from different professional backgrounds and work experience enrolled in the programs, thus reinforcing a culture of interprofessional practice. Both programs were delivered in a way that created a climate within which the contributions of educators from different disciplines were acknowledged and valued.

In terms of mode of delivery, the programs used blended and fully online learning to make the courses flexible and available off-campus, acknowledging the busy work schedules and needs of 21st-century health professionals who are unlikely to commit their time for fully face-to-face courses.

Regarding teaching and learning strategies, the successful creation of learning communities in faculty development programs has been described as utilizing diverse educational methods grounded in adult learning theories, including experiential learning and peer learning, reflection, and feedback [5,8]. Therefore, both programs’ learning activities were designed to facilitate critical thinking, collaboration and interaction, practical applications to their educational practice, and workplace-based reflection and learning. This facilitated inclusivity and promoted learning and acknowledgment of the educational practices of each other’s healthcare disciplines, supporting participants’ roles as educators in health and social care environments in which professional collaboration is increasingly essential.

The design and delivery of the programs attracted a multidisciplinary mix of educators who participated and interacted with each other throughout the different cohorts. Table 2 shows the diversity of health professions involved in the cohorts from 2018 to 2021. The interprofessional diversity in the core academic teams also provided the opportunity to model interprofessional teaching and facilitation of learning, which was enhanced by the associated tutors from health and non-health-related disciplines who taught and graded across the different modules.

The educators and the core academic teams reported positive feedback across both programs’ internal quality assurance reports and from students’ evaluation surveys. For the postgraduate diploma in health professions education at the University of the Andes, 91% (n=111) of students expressed being overall very satisfied with the course and 86% (n=111) expressed being very satisfied with the course's collaborative and interactive features. For the clinical education program at the University of East Anglia, students’ qualitative comments reflected their satisfaction with the online learning environment, the approachability and presence of the core academic team, and the applicability and interactive nature of the learning activities. The clinical education program also received external validation in the form of being accredited for the Fellowship of the Higher Education Academy and Membership of the Academy of Medical Educators. This shows an alignment with the UK Professional Standards Framework for Teaching and Learning [9] and with the Professional Standards for Medical Educators set by the Academy of Medical Educators [10].

## Lessons learned

Program leaders across both courses have jointly reflected and agreed that the creation of an interprofessional environment was mainly facilitated by 3 features: (1) a professions-inclusive teaching and facilitating style, (2) a flexible learning climate, and (3) interprofessional peer work. These constitute the main reflection points and lessons learned from the experience of planning and delivering these online IPE faculty development programs for educational practice improvement, which may well be transferable to other faculty development programs seeking to enhance and support the interprofessionality of their courses.

Regarding a professions-inclusive teaching style, the interprofessional setting was facilitated by designing neutral online resources and not favoring any healthcare discipline over the others. Teaching and facilitation were conducted through fully online and blended learning activities, where the core academic teams delivered tutorials using a mix of examples, resources, applications, and guided readings of the literature from different health professions and focused on topics that provided common ground, such as clinical and student-centered teaching. The multidisciplinary core academic teams encouraged educators to appreciate each other’s backgrounds so that all participants gained the best experience from the courses. This constitutes an essential part of the delivery of the interprofessional hidden curriculum, as previous research has shown that learners report clinical faculty behaviors as influencing their own practices and, therefore, could promote interprofessional practices in their own educational settings [5,11].

Concerning the development of a flexible learning environment, it was essential to implement personalized tutorials, online office hour meetings, and catch-up recordings of sessions to support educators’ needs and personal interests. Furthermore, planning a reasonable workload that suited busy health professionals, flexible attendance and assignment submission, and balanced face-to-face/synchronous and asynchronous contact helped to support the engagement of a mix of clinicians with demanding schedules. These practices have been reported as desirable for online learning programs’ success, especially in light of the abrupt transition to online education as a result of the COVID-19 pandemic [6,7].

Finally, interprofessional peer work was conducted by employing on-campus and online discussions, debates, and teamwork, with a balance of professional membership in groups, and through different peer-assessment activities in which the core academic teams deliberately conducted interprofessional matching. As IPE involves learning ‘with, from and about’ and it is not just a mix of people acquiring the same knowledge or developing the same educational skills, programs should be designed to include abundant opportunities for interaction and exchange [1,2]. This may facilitate the readiness of educators to work and seek opportunities for interprofessional collaboration in their daily practice.

The implementation of educational practices incorporating these lessons learned from both faculty development programs is expected to play a unique role in promoting IPE, as these lessons address the major barriers to teaching and learning described above at both the individual and organizational level, with the added value of providing educators with the knowledge and skills needed to design and facilitate IPE as part of their own educational role.

## Conclusion

Faculty development initiatives that seek to prepare educators for interprofessional learning and collaborative work should be an integral component of health professions education, as delivering these courses within professional silos is no longer justifiable. The successful implementation and delivery of these 2 programs, in culturally diverse settings and with similar experiences across both institutions, showed that IPE was facilitated by creating an environment conducive to collaborative working and learning from each other. Course developers carefully considered the pedagogical approaches used, the learning environment created, and the use of social learning to support communities of learning and interprofessional collaboration. This enabled educators from various professional backgrounds to learn together and from each other, with the support of a flexible and adaptable core academic team able to collaborate and continually learn about, from, and with each other. The latter was possible partly due to the values and practices brought by the multidisciplinary teaching teams and the programs’ structure. Future research should explore and evaluate the perception and effectiveness of these interprofessional settings for educators, along with potential transferability to their teaching practices. As the relevance of IPE grows, an effective way of promoting it is to train the trainers in formal and explicit interprofessional settings.

## Figures and Tables

**Table 1.** General overview of the structure of both programs

	Clinical education (master’s-PG diploma-PG certificate)	PG diploma in health professions education
	University of East Anglia (UK)	University of the Andes (Chile)
	Since 2012–2013 (9 cohorts)	Since 2018–2019 (3 cohorts)
Purpose	Enhance and refresh clinical teachers’ educational practices in an interprofessional learning environment	Enhance and refresh clinical teachers’ educational practices in an interprofessional learning environment
Learners	Clinical educators from multiple health-related disciplines	Clinical educators from multiple health-related disciplines
Academic team	A multidisciplinary team of tutors	A multidisciplinary team of tutors
Mode of delivery	Blended learning (face-to-face and online learning options)	Online learning
Curricular structure	Modular with a core & options component	Modular
Teaching & learning components	Learning & teaching, assessment, curriculum, and management & leadership in health professions education	Learning & teaching, assessment, curriculum, and management & leadership in health professions education
Research components	Systematic reviews, quantitative and qualitative research modules, and a master’s dissertation	None
Online learning activities	Synchronous and asynchronous activities: online tutorials/eWorkshops, videos, guided readings, live workshops and seminars, discussion boards/blog activities, student-led activities and presentations, one-to-one tutorials and guidance.	Synchronous and asynchronous activities: online tutorials/eWorkshops, videos, guided readings, live workshops and seminars, discussion boards/blog activities, student-led activities and presentations, one-to-one tutorials and guidance.
Online assessment activities	Formative and summative: essays, presentations, formative quizzes, lesson plans, written exams, critical appraisal of the literature, work-based project, research proposals, and submission of a research dissertation.	Formative and summative: discussion board entries, reflective portfolios, formative quizzes, peer and self-assessment activities, presentations and essays.
Focus	International	Regional (Latin America)
Language of delivery	English	Spanish

PG, postgraduate.

**Table 2.** Interprofessional student and staff profile of both programs (2018–2021)^a)^

	Student profile		Core academic team profile	
	Clinical education (master’s-PG diploma-PG certificate)	PG diploma in health professions education	Clinical education (master’s-PG diploma-PG certificate)	PG diploma in health professions education
	University of East Anglia (UK)	University of the Andes (Chile)	University of East Anglia (UK)	University of the Andes (Chile)
Assistant practitioners	1	0	0	0
Biological sciences	0	0	1	0
Dentists	1	54	1	4
Dietitians	0	4	0	0
Midwives	0	1	1	0
Medical technologist	0	1	0	0
MBBS students (intercalating degree)	51	0	-	-
Nurses	23	11	1	1
Occupational therapists	1	1	0	0
Paramedics	2	0	1	0
Pharmacists	1	0	0	0
Physical therapists	2	6	0	1
Physicians	46	32	2	0
Veterinarians	0	1	0	0
Total participants	128	111	7	6

PG, postgraduate.

a) Student and staff profiles are presented from 2018 to 2021 in order to make data comparable.

## Supplementary materials

Data files are available from Harvard Dataverse: https://doi.org/10.7910/DVN/UEIGWO

Supplement 1. Clinical education (master’s, postgraduate diploma, and postgraduate certificate) course program/handbook.

Supplement 2. Postgraduate diploma in health professions education course program/handbook.

Supplement 3. Audio recording of the abstract.
