# Supplementary material for: Implementation and lessons learned from 2 online interprofessional faculty development programs for improving educational practice in the health professions in Chile and the United Kingdom from 2018 to 2021
Source: J Educ Eval Health Prof. 2021 Aug 9;18:21. doi: 10.3352/jeehp.2021.18.21 (PMC8441097; doi:10.3352/jeehp.2021.18.21)
Supplement: Supplementary file 1 — Supplement 1. Clinical education (master’s, postgraduate diploma, and postgraduate certificate) course program/handbook. [file jeehp-18-21-suppl1.pdf]

University of East Anglia | NORWICH MEDICAL SCHOOL

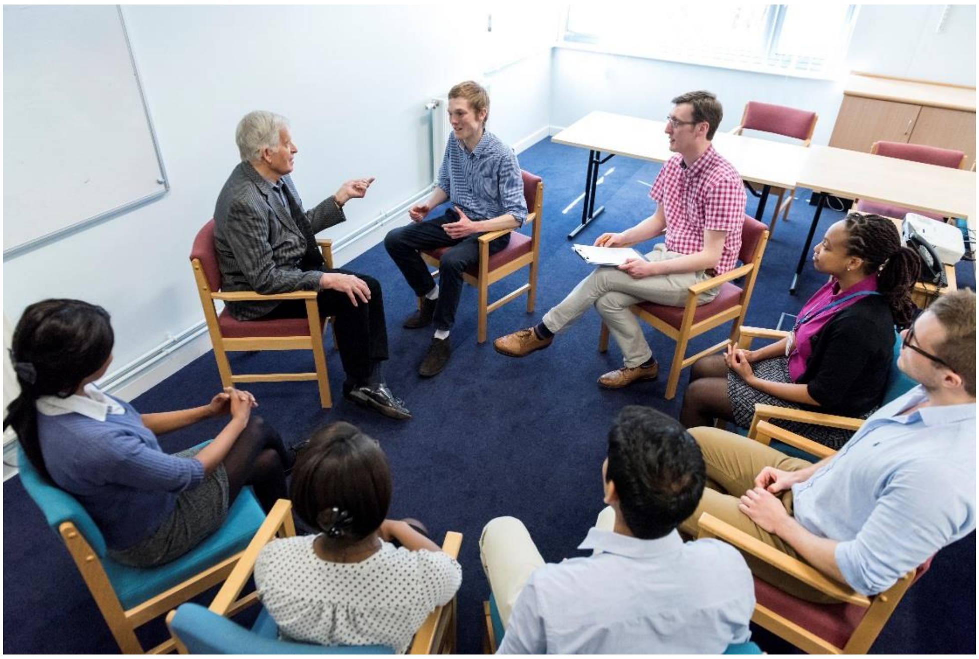

PGCERT/ PGDIP/MCLINED STUDENT HANDBOOK 2020-2021

AdvanceHE

Accredited by
Advance HE
&
Academy of Medical Educators

AoME ACADEMY OF MEDICAL EDUCATORS

© Copyright University of East Anglia, June 2016

This material may not be reproduced in whole or in part without the written permission of the Head of the School of Health Sciences of the University of East Anglia

Contents

1.0 INTRODUCTION 3
1.1 Welcome Message from the Course Director 3
1.2 UEA Student Handbook and your Faculty and Course Handbooks 4
2.0 CLINICAL EDUCATION PROGRAMME STRUCTURE, CONTENT AND ARRANGEMENTS 4
2.1 Description of the Clinical Education Programme 4
2.2 Educational Aims of the Programme 5
2.3 Values and Ethos of the Programme 8
2.4 Attendance and Engagement 9
2.5 Compulsory and Optional Modules on the Postgraduate Certificate, Postgraduate Diploma and Masters Programmes 10
2.6 Module Descriptors 11
2.6.1 Module Descriptors for PGCert – Standard/ Online Mode 11
2.6.2 Module Descriptors for PGCert – Primary Care Tutor Mode 20
2.6.3 Module Descriptors for PGDip and Masters Modules 28
2.7 Calendar of Modules Available within the Clinical Education Programme 34
PART TIME STUDY 34
FULL TIME STUDY 37
2.8 Students Returning to MBBS Studies following MClinEd Completion 38
2.9 Access to Work Based Opportunities for Clinical Education Programme studies 38
2.10 Pastoral Care and Academic Advice for Students on the Clinical Education Programme 38
2.11 Clinical Education Programme Module Organisers 39
2.12 New Appointments of FMH Lecturers 40
2.13 Professional Recognition (Associate Fellowship/ Fellowship of HEA) 40
2.13.1 Frequently asked questions 40
2.13.2 Professional Development Log 43
2.13.3 Observation of Teaching Form 47
2.13.4 Professional Development Log Assessment Form for HEA Recognition Status 56
2.13.5 HEA Professional Recognition Judgements & Quality Assurance Process 59
2.14 Professional Recognition (Membership of the Academy of Medical Educators) 60
APPENDICES 61
APPENDIX I: ASSESSMENT GUIDELINES 62
APPENDIX II: MARKING OF STUDENT WORK 68
APPENDIX III: LEARNING CONTRACT FOR WORK-BASED PROJECT 70

1.0 INTRODUCTION

1.1 Welcome Message from the Course Director

Dear Student,

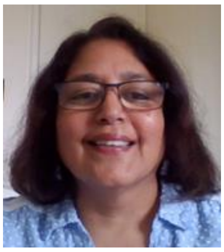

I am delighted to welcome you to the PGCert/ PGDip/ Master of Clinical Education programme at the University of East Anglia (UEA).

Contributing to the educational and professional development of students and junior colleagues is a professional requirement of all healthcare professions. The Postgraduate Taught Programme in Clinical Education offers health and social care professionals the opportunity to develop and enhance their skills in teaching, supervising and supporting the learning of others in clinical practice and/or higher education.

This programme is a collaborative initiative between the School of Health Sciences and the Norwich Medical School, within the Faculty of Medicine and Health Sciences (FMH), UEA. Building on the existing reputation of the Faculty, this course offers you the ideal opportunity to learn from experienced clinical educators in an inter-professional context, using a blended approach consisting of workshops and distance learning. Course activities are designed to facilitate critical thinking, collaboration, workplace-based reflection and learning, and your professional development as an educator.

The PGCertClinEd is delivered in three modes: a standard (face to face) mode delivered part-time over one year, an online mode also delivered part-time over one year, and an alternative mode for primary care tutors who provide clinical teaching to our medical students. The three modes are delivered separately, although the learning outcomes, themes, and assessments for all modes remain the same. The content and delivery of the programme complements relevant Professional, Statutory and Regulatory Body's (PSRB) standards and frameworks and is aligned to the UK Professional Standards Framework for Teaching and Supporting Learning in Higher Education (UKPSF, 2011). All modes of the course are accredited for professional recognition by Advance HE at D1/D2 levels (AFHEA/FHEA) and for membership (MAcadMEd) of the Academy of Medical Educators.

Due to the social distancing required because of the COVID pandemic, we have taken the decision to offer the two online modules of the PGCertClinEd (HSC-7165D & HSC-7166H) instead of the face to face modules (HSC-7065D & HSC-7066H), combining synchronous and asynchronous teaching methods to ensure you get the best out of this course. For students undertaking the full time Masters/ Diploma, the research methods modules will also be delivered remotely unless national guidance on social distancing changes. We will deliver any synchronous/ remote teaching on the days when your presence would normally have been required on campus, so that you are not inconvenienced in any way. Further information will be provided within each module.

I hope you find this course fulfilling and rewarding. If you have any queries or need further information, please do not hesitate to contact me, the module organisers or your personal adviser.

I look forward to meeting you in due course.

Yours sincerely

Professor Veena Rodrigues MBBS, MD, MPhil, FFPH, MClinEd, FAcadMEd, SFHEA

Course Director

E-mail: v.rodrigues@uea.ac.uk Twitter: @vcrodrigues_uea

1.2 UEA Student Handbook and your Faculty and Course Handbooks
The central UEA student handbook which contains information about all key UEA policies and guidance can be found on the UEA Portal:
https://www.uea.ac.uk/learningandteaching/getting-started/handbooks

The central UEA student handbook is for all students studying on undergraduate and postgraduate taught programmes. It aims to give new and continuing students a central reference point for University-wide regulations, processes and guidance to help and support you through your studies. Useful information is also available on the "I ♥ Health" Blackboard site which you will have access to via the Portal Home tab.

All information within this handbook is specific only to your course **– Masters, Postgraduate Diploma and Postgraduate Certificate in Clinical Education.**

2.0 CLINICAL EDUCATION PROGRAMME STRUCTURE, CONTENT AND ARRANGEMENTS

2.1 Description of the Clinical Education Programme
The Postgraduate Taught Programme in Clinical Education offers all health and social care professionals the opportunity to develop their skills in teaching, assessing, supervising and supporting the learning of others in clinical practice and/or higher education.

The programme has been developed to suit the needs of qualified and trainee doctors, nurses, midwives, public health nurses and all allied health professionals, who wish to follow either a part time or full time programme of study. In addition, with line managers' permission, the programme may also be used by employees of the UEA as an alternative to the PGCERT-HEP as part of the probationary requirement for teaching staff (see section 2.12).

The Postgraduate Taught Programme in Clinical Education is delivered jointly by the School of Health Sciences (HSC) and the Norwich Medical School (MED) and builds on the existing reputation of the Faculty of Medicine and Health Sciences for interprofessional teaching and learning, promoting learning that will equip healthcare professionals for their roles as educators in health and social care environments, where professional collaboration is increasingly essential. This learning experience is enhanced by the diversity of professionals who contribute to the teaching, and the students enrolled on the programme, which reinforces the culture of interprofessional practice.

Students of the programme will explore advances in both theory and practice in clinical education and apply their knowledge through practical design and development of teaching and inquiry methods. The modules will encourage your reflection on your own learning in order to support learning in others, developing the skills to design teaching, which will be applied to your own discipline and clinical areas.

Through the programme, students can opt to undertake studies leading to:

- Postgraduate Certificate in Clinical Education (PGCertClinEd) – 60 M-level credits which can be completed by undertaking two compulsory modules from the first year of the part time Master's programme. Successful completion of these modules meets the UK 'Professional Standards Framework for teaching and supporting learning in higher education' (see section 2.2) and confers eligibility for Fellowship of the Higher Education Academy (HEA).

OR

- Postgraduate Diploma in Clinical Education (PGDipClinEd) – 120 M-level credits, which can be completed by undertaking modules from the first two years of the three-year, part time Master's programme, or alternatively by taking these modules over the course of one year's full time study.
  NB. Completion of the Postgraduate Diploma in Clinical Education will involve students in completing the components of the Postgraduate Certificate in Education and so, will also confer eligibility for Fellowship of the HEA.

OR

- Master of Clinical Education (MClinEd) – 180 M-level credits, which can be completed through three years of part time study or through one year of fulltime study.
  NB. Completion of the Master of Clinical Education will involve students in completing the components of the Postgraduate Certificate in Education and so, will also confer eligibility for Fellowship of the HEA.

For the award of PGCertClinEd, PGDipClinEd and MClinEd, each 20credit module usually involves approximately 200 effort hours, and 40credit module approximately 400 effort hours. This includes hours spent on online activities, synchronous and asynchronous teaching, pre- and post-class activities, independent study, workplace based learning and reflection, as well as time required to prepare assignments.

Details of the compulsory and optional modules that students will complete to gain the award of Masters, Postgraduate Diploma or Postgraduate Certificate are provided in Section 2.5.

2.2 Educational Aims of the Programme

The Clinical Education programmes seek to provide a vehicle for health and social care professionals to develop their skills for teaching and support for learning in clinical practice and/or higher education. To that end, the content and delivery of programmes closely articulate with the UK Professional Standards Framework for Teaching and Supporting Learning in Higher Education (UKPSF).

At the heart of this framework is acknowledgement of the distinctive nature of teaching in higher education, respect for the autonomy of higher education institutions, and recognition of the sector's understanding of quality enhancement for improving student learning. The framework recognises that the scholarly nature of subject inquiry and knowledge creation, and a scholarly approach to pedagogy, combine to represent a unique feature of support for student learning in higher education institutions.

The UK Professional Standards Framework provides a general description of the main dimensions of the roles of teaching and supporting learning within the HE environment. These are a set of statements outlining the **Areas of Activity** undertaken by teachers and supporters of learning within Higher Education, the **Core Knowledge** that is needed to carry out those activities at the appropriate level, and the **Professional Values** that someone performing these activities should embrace and exemplify.

Areas of Activity (A)

1. Design and plan learning activities and/or programmes of study.
2. Teach and/or support learning.
3. Assess and give feedback to learners.
4. Develop effective learning environments and approaches to student support and guidance.
5. Engage in continuing professional developments in subjects/disciplines and their pedagogy, incorporating research, scholarship and the evaluation of professional practices.

Core Knowledge (K)

1. The subject material.
2. Appropriate methods for teaching and learning in the subject area and at the level of the academic programme.
3. How students learn, both generally and within their subject/discipline area?
4. The use and value of appropriate learning technologies.
5. Methods for evaluating the effectiveness of teaching.
6. The implications of quality assurance and quality enhancement for academic and professional practice with a particular focus on teaching.

Professional Values (V)

1. Respect individual learners and diverse learning communities
2. Promote participation in higher education and equality of opportunity for learners
3. Use evidence informed approaches and the outcomes from research, scholarship and continuing professional development
4. Acknowledges the wider context in which higher education operates recognising the implications for professional practice.

Source: Higher Education Academy (2011) The UK Professional Standards Framework for teaching and learning in higher education. http://www.heacademy.ac.uk/ukpsf

Programme Outcomes

With these professional standards in mind, the clinical education programmes (Postgraduate Certificate, Postgraduate Diploma and Masters) have been developed to satisfy a range of outcomes as detailed overleaf.

<u>For the PG CERTIFICATE in Clinical Education</u> *(for pathways see Section 2.7)*

By the end of the PG Certificate students will be able to:

1. Design and plan learning activities appropriate to their workplace and role. (A1)
2. Discuss and apply their knowledge and skills in teaching and/or supporting other adult learners. (A2)
3. Create and implement assessment and the provision of feedback, *which are appropriate to their workplace and role.* (A3)
4. Develop effective learning environments with support and guidance for other adult learners. (A4)
5. Engage with continuing professional development in their professional discipline within a framework of reflection, incorporating research, scholarship and evaluation of professional practice. (A5)
6. Research and appraise relevant subject material and educational theory in educational practice. (K1)
7. Apply and critique methods of teaching, learning and assessment appropriate to their workplace and role. (K2)
8. Explore own and other adult learners learning styles and preferences within their discipline. (K3)
9. Use and discuss the value of a range of learning technologies. (K4)
10. Implement and review methods for evaluating the effectiveness of teaching and appreciate how session/course/curriculum evaluation informs future planning and delivery. (K5)
11. Consider the implications of quality assurance and quality enhancement or academic and professional practice with a particular focus on teaching. (K6)
12. Demonstrate and reflect upon the following professional values (V1-4):
    - Respect individual learners and diverse learning communities
    - Promote participation in higher education and equality of opportunity for learners
    - Use evidence-informed approaches and the outcomes from research, scholarship and continuing professional development
    - Acknowledge the wider context in which higher education operates recognising the implications for professional practice.

The compulsory modules of the Postgraduate Certificate in Clinical Education have been mapped against the UKPSF (see letters and numbers in brackets above. Students who undertake these modules are eligible to apply for professional recognition as Associate Fellows/ Fellows of the Higher Education Academy (see Section 2.13 for further details on the application process). These modules have also been accredited for membership of the Academy of Medical Educators (AoME).

For the PG DIPLOMA in Clinical Education *(for pathways see Section 2.7)*

By the end of the PG Diploma programme students will have:

1. Reinforced skills and understandings outlined for PG Certificate;
2. Had an opportunity to further develop some of the practical skills and understandings, according to the optional modules they selected;
3. Begun to develop their skills of critical appraisal of research evidence and theory; and begun to apply their critical understanding to education practice.

For the MASTER of Clinical Education *(for pathways see Section 2.7)*

By the end of the Master's programme students will:

1. Be able to demonstrate the practical skills and understandings outlined for the PG Certificate and the PG Diploma;
2. Have further honed their skills of critical appraisal and their ability to synthesise findings of research and apply their findings to education practice;
3. Have developed their research skills through the conduct of a research dissertation, demonstrating their ability to identify an appropriate research question and to design research to answer that question;
4. Have extended their knowledge of education research through successful completion of a research dissertation.

2.3 Values and Ethos of the Programme

The ethos or philosophy of the Clinical Education programme is quite distinctive, partly because of the focus of the programme and partly as a result of the values brought to it by the interprofessional teaching team. The following values should be strongly evident within the programme and there is an expectation that students of the programme will also observe these values in their own practice:

- **Adult Learning** – the educational philosophy and practice that will be observed on the programme will be that of adult learning. This expects that individual students consider their own learning needs and seek to service these, as far as is possible within the formal award bearing structures of the programme, by working in active collaboration with teaching staff and fellow students. Teaching staff will model negotiation (where appropriate) and as the programme progresses, there will be a shift from lecturers 'teaching' to the facilitation of learning.
- **'Masterliness'** – it is expected that students engaging in this programme will themselves be seeking to study at an appropriate level for Masters. The Quality Assurance Agency's Framework for Higher Education Qualifications describes what is expected of students that achieve Masters level studies, as follows:
  - a systematic understanding of knowledge, and a critical awareness of current problems and/or new insights, much of which is at, or informed by, the forefront of their academic discipline, field of study or area of professional practice
  - a comprehensive understanding of techniques applicable to their own research or advanced scholarship
  - originality in the application of knowledge, together with a practical understanding of how established techniques of research and enquiry are used to create and interpret knowledge in the discipline
  - conceptual understanding that enables the student to:
    - evaluate critically current research and advanced scholarship in the discipline
    - evaluate methodologies and develop critiques of them and, where appropriate, to propose new hypotheses.

- **Integrity** – it is expected that both staff and students of the programme will behave with integrity (the dictionary definition of this is that they will be demonstrating rightness of behaviour, honour and good virtue). One example of where integrity might be important within the programme pertains to the tone in which past and present learning experiences are described. There are a number of junctures in the programme where students and staff engage in analysis of their past and ongoing learning experiences. During the course of such events, it is expected that individuals would not use discussion as a vehicle to overtly criticise professional colleagues or programmes. Instead, there is an expectation that a professional tone would be adopted in discussion of past negative and positive learning/teaching experiences and that such discussions will remain confidential to the group.
- **Tolerance** – the programme team consider that tolerance of others, especially in the classroom, is essential to maximise learning for all. Students are expected to demonstrate tolerance of the views of each other, fellow students' learning styles and needs and the range of different teaching styles, devices and practices that they will be exposed to during the course of the programme.
- **Curiosity** - students may well be faced with new and uncomfortable situations and learning methods (e.g. student-centred activities, student led learning and the use of novel resources including new IT software and Blackboard facilities). The team expect students to demonstrate a willingness and interest in trying new ways of learning and teaching.
- **Egalitarianism** – the programme team are committed to ensuring that all students are given equal rights and opportunities, irrespective of their profession, professional seniority or academic abilities. Because the programme is open to both qualified health and social care professionals and intercalating MBBS students, differences in professional standing outside the boundaries of the course may be quite stark. However, it is expected that within the confines of the course, all students should regard each other as equal and fellow learners. Upon completion of the MClinEd, some special arrangements may be necessary for students returning to MBBS studies, especially if they might be taught by qualified professionals who have also been students of the MClinEd (see section 2.8).

2.4 Attendance and Engagement
https://portal.uea.ac.uk/learning-and-teaching/students/the-basics/absence
In addition to the UEA requirement for 80% attendance over the course, because of the specific nature of the course contact days in some modules, and the experiential learning included, students will be required to achieve at least 60% attendance in each module. Students who are unable to participate in the module activities are required to request absence via the link above through e:Vision, which is then referred to the Course Director for consideration. You might be asked **to** provide evidence to support the request. It will be the student's responsibility to make up for any lost learning opportunities as a result of absence. Students with excessive absences might be required to interrupt studies and re-take the module in the following year. Within the online modules, participation in synchronous sessions and engagement with the discussion boards and online activities will form the attendance requirement.

We actively encourage feedback from students in order to continually improve our course content, delivery, assessment and organisation. Within the three education modules, learner feedback is collected mid-module so that we can respond to any student issues or concerns swiftly. All modules also seek feedback through end of module evaluations from students.

2.5 Compulsory and Optional Modules on the Postgraduate Certificate, Postgraduate Diploma and Masters Programmes

**For the award of PGCertClinEd**
Students must successfully complete the two compulsory modules below:<br>**Standard/ Online Mode:**<br>1. Enhancing Teaching Learning and Assessment in Clinical Education<br>2. Leading Teaching Learning and Assessment in Clinical Education<br>**Primary Care Tutor Mode:**<br>1. Clinical Supervisor<br>2. Educational Supervisor
**For the award of PGDipClinEd**
Students must successfully complete the four compulsory modules below:<br>**Standard/ Online Mode:**<br>1. Enhancing Teaching Learning and Assessment in Clinical Education<br>2. Leading Teaching Learning and Assessment in Clinical Education<br>3. Leading Contemporary Clinical Education<br>4. Introduction to Research Methods<br>**Primary Care Tutor Mode:**<br>1. Clinical Supervisor<br>2. Educational Supervisor<br>3. Leading Contemporary Clinical Education<br>4. Introduction to Research Methods<br>**AND** must then successfully complete **one** further 20 credit, taught research module:<br>• Further Quantitative Research Methods<br>OR<br>• Further Qualitative Research Methods<br>OR<br>• Systematic Reviews and Research Synthesis
**For the award of MClinEd**
Students must complete the four compulsory modules below:<br>**Standard/ Online Mode:**<br>1. Enhancing Teaching Learning and Assessment in Clinical Education<br>2. Leading Teaching Learning and Assessment in Clinical Education<br>3. Leading Contemporary Clinical Education<br>4. Introduction to Research Methods<br>**Primary Care Tutor Mode:**<br>1. Clinical Supervisor<br>2. Educational Supervisor<br>3. Leading Contemporary Clinical Education<br>4. Introduction to Research Methods<br>**AND** must then successfully complete **one** further 20 credit, taught research module:<br>• Further Quantitative Research Methods<br>OR<br>• Further Qualitative Research Methods<br>OR<br>• Systematic Reviews and Research Synthesis<br>**AND**<br>• The 60 credit DISSERTATION

2.6 Module Descriptors
This section provides descriptors for each of the modules outlined previously. The module organisers may also provide you with additional material to support your learning.

Section 2.7 shows the availability of compulsory and optional modules during the year so that students can see which modules are available when.

2.6.1 Module Descriptors for PGCert – Standard/ Online Mode

**Students opting to study for the award of a Postgraduate Certificate in Clinical Education must successfully complete the TWO following <u>COMPULSORY</u> 20 credit and 40 credit modules. Successful completion of the PGCert modules will confer eligibility to apply professional recognition status at D1/D2 (AFHEA/FHEA) of UKPSF, 2011**

HSC-7165D ENHANCING TEACHING, LEARNING AND ASSESSMENT IN CLINICAL EDUCATION
(COMPULSORY; 20 credits)

MODULE OVERVIEW
This module will introduce students to the underpinning educational theory and practice required to enhance their clinical education role, particularly as a clinical supervisor, mentor, or practice educator. The module recognises the uniqueness of learning in clinical and practice settings and the importance of the role of the educator in facilitating learning which occurs in the context of client care. Learners develop skills and experience professional socialisation which cannot readily be acquired elsewhere.

The overall aim of the module is to provide educators with a body of knowledge and principles to facilitate teaching, learning and assessment in practice-based settings. This will enable the educator to work effectively with learners who will become the workforce of the future and ultimately to improve services.

This module is relevant to all health and social care professionals who supervise/mentor students, other clinicians who teach in higher education, whether they are new to these roles or new to studying at Masters level.

MODULE STRUCTURE/DELIVERY
This module is structured around four themed blocks with content released fortnightly. Each block will contain a combination of synchronous and asynchronous activities following best practice in online pedagogies. Synchronous sessions will be delivered on the day when your presence would have been required on campus, so that you are not inconvenienced. All asynchronous sessions can be completed at your own pace within the fortnightly block release dates. Each block will contain suggestions for workplace based learning and reflection.

The four themes are:

- The learning environment in clinical education
- Facilitation of learning
- Assessment and evaluation in clinical education.
- Self-development in clinical education

Block 1: The learning environment in clinical education
This block will explore the importance of an effective learning environment in order that learners can progress to meet defined learning outcomes. It will include how to establish and maintain an effective educator/learner relationship in the workplace as well as motivation and basic psychological needs of learners.

Block 2: Facilitation of Learning
This block will cover some of the key principles of facilitation of learning. Block 1 established that the relationship between the educator and the learner is fundamental to facilitation of learning. Block 2 will include educational theories and how these apply to learning in the workplace. We will also cover lesson planning and various types of teaching methods to facilitate learning.

Block 3: Assessment and evaluation in clinical education
This block covers the principles of assessment in the context of healthcare practice, the concept of competency and how this relates to practice assessment. It will also cover identifying and supporting the failing student, giving effective feedback and creating robust plans to manage the situation. We will touch on evaluation of learning which will be further developed in block 4.

Block 4: Self-development in clinical education
This block provides an opportunity to look into reflection and reflective practice, and the role it plays in continuing professional development of healthcare professionals. It will also cover peer observation and how individuals can evaluate their role and responsibilities as an educator.

In addition, the module will include self-directed reading, case scenarios and discussion, synchronous sessions with group work, opportunities for online discussions, and suggestions for workplace-based reflection and learning. Students are strongly encouraged to arrange a peer observation of face to face or online teaching or to submit a video recording of a teaching session they have delivered in order to receive formative feedback on their teaching skills, and thus authenticate their educator practice. This is an essential part of your application for Fellowship of HEA. Details will be provided by the module organiser and/or the professional recognition lead prior to these activities.

MODULE OUTCOMES
At the end of this module the student will be able to:

- Design and plan learning activities appropriate to their workplace and role.
- Discuss and apply their knowledge and skills in teaching and/or supporting other adult learners.
- Consider and implement assessment and the provision of feedback to learners, which are appropriate to their workplace and role.
- Develop effective learning environments with support and guidance for other adult learners.
- Engage with continuing professional development in their professional discipline within a framework of reflection, incorporating research, scholarship and evaluation of professional practice.
- Research and appraise relevant subject material and educational theory in educational practice.
- Apply and critique methods of teaching, learning and assessment appropriate to their workplace and role as an educator.
- Explore own and other adult learners learning styles and preferences within their discipline.
- Implement and review methods for evaluating the effectiveness of teaching and appreciate how evaluation informs future planning and delivery.

- Demonstrate and reflect upon the following professional values:
    - Respect individual learners and diverse learning communities
    - Use evidence-informed approaches and the outcomes from research, scholarship and continuing professional development.

Midwives and nurses only
In May 2018 the Nursing and Midwifery Council published Part 1: Standards framework for nursing and midwifery education and Part 2: Standards for student supervision and assessment. This module has been validated as a route to prepare midwives and nurses for the roles of practice supervisor and practice assessor at level 7.

MODULE ASSESSMENT
Formative Mid-module Assessment
You will be required to submit a 1,000 word reflective essay critically evaluating your development as an educator. You will receive a mark and written feedback from a tutor to help you to understand how to approach your summative assessment.

4000-wordEnd of Module Summative Assessment
Assignment title: An evaluation of your development as an educator.

This assignment is designed to allow you to critically evaluate your personal and professional development in relation to the role and responsibilities of the educator. You are required to provide a 4000-word evaluation of your development as an educator; this should clearly demonstrate your ability to critically appraise relevant underpinning educational theory and evidence.

You will be required to demonstrate:

1. Critical analysis of the factors that influence how learners integrate into clinical/educational practice settings, and are able to meet defined learning outcomes
2. Critical evaluation of the necessary teaching and learning methods in clinical/educational practice settings in order to meet the needs of learners
3. Critical evaluation of the assessment process, including the provision of effective feedback to learners
4. Critical reflection of self and peer evaluation to facilitate continuing professional development in the role of the educator
5. Critical appraisal of relevant subject material and educational theory in educational practice.

Submission Date: January (refer to e-vision for date)

Professional Recognition
This module has been accredited for Associate Fellowship of HEA (AFHEA) by Advance HE. Students completing this module will be encouraged to compile a portfolio of evidence against the UKPSF if they wish to apply for AFHEA status. The application requires completion of a professional development log and a teaching observation report OR two testimonials from professional colleagues familiar with your educational activities. Please refer to section 2.13 for further details on the application process for professional recognition.

ARTICULATION OF MODULE LEARNING OUTCOMES WITH UK PSF (2011)

Module Learning Outcomes<br><br>At the end of the module the student will be able to:	UK PSF Areas of Activity, Core Knowledge and Professional Values (2011)
Design and plan learning activities appropriate to their workplace and role.	Areas of Activity: 1, 3, 4, 5,<br><br>Core Knowledge: 1, 3, 4,<br><br>Professional Values: 1, 2, 3, 4,
Discuss and apply their knowledge and skills in teaching and/or supporting other adult learners.	Areas of Activity: 2<br><br>Core Knowledge: 1, 2, 3,<br><br>Professional Values: 1, 2
Consider and implement assessment and the provision of feedback to learners, which are appropriate to their workplace and role.	Areas of Activity: 3, 4, 5,<br><br>Core Knowledge: 2, 3, 5, 6<br><br>Professional Values: 1, 3
Develop effective learning environments with support and guidance for other adult learners.	Areas of Activity: 2, 4,<br><br>Core Knowledge: 1, 2, 3, 4<br><br>Professional Values: 1, 2, 3, 4
Engage with continuing professional development in their professional discipline within a framework of reflection, incorporating research, scholarship and evaluation of professional practice.	Areas of Activity: 5<br><br>Core Knowledge: 1, 5, 6<br><br>Professional Values: 3, 4
Research and appraise relevant subject material and educational theory in educational practice.	Areas of Activity: 5<br><br>Core Knowledge: 5, 6<br><br>Professional Values: 3, 4
Apply and critique methods of teaching, learning and assessment appropriate to their workplace and role as an educator.	Areas of activity: 2, 5<br><br>Core Knowledge: 1, 2, 3,5 ,6<br><br>Professional Values: 1,3, 4
Explore own and other adult learners learning styles and preferences within their discipline.	Areas of Activity: 5<br><br>Core Knowledge: 2, 3,<br><br>Professional Values: 1, 3
Implement and review methods for evaluating the effectiveness of teaching and appreciate how evaluation informs future planning and delivery.	Areas of Activity: 2, 5<br><br>Core Knowledge: 5<br><br>Professional Values: 3

Demonstrate and reflect upon the following professional values;<br><br>• respect individual learners and diverse learning communities<br>• use evidence - informed approaches and the outcomes from research, scholarship and continuing professional development	Areas of Activity: 2, 5<br><br>Core Knowledge: 2, 3,<br><br>Professional Values: 1, 2, 3, 4
**Midwives Only** - As a sign-off mentor confirm and justify the decision that students have met, or not met, the NMC (2009) *Standards for preregistration midwifery education* and are capable of safe and effective practice.	Areas of activity: 2, 3, 5<br><br>Core Knowledge: 2<br><br>Professional Values: 3

HSC-7166H LEADING TEACHING, LEARNING AND ASSESSMENT IN CLINICAL EDUCATION
(Compulsory; 40 credits)

MODULE OVERVIEW
This module is relevant to all health and social care professionals who supervise/mentor students; support others as educators and/or lead educational activities in the clinical and or higher education environment. The module aims to enhance your clinical education leadership through further developing your teaching, learning and assessment roles, and considers the changing healthcare contexts and challenges applicable to leaders in health care education. Part of this will include undertaking an educational improvement project.

The module offers those with professional experience the opportunity to pursue postgraduate education relevant to their interests and requirements to develop their educational leadership role. The module enables participants to explore work based opportunities with theoretical underpinning to enhance their academic practice as a result. Participants will gain a comprehensive understanding of how to manage the learning of others, whilst developing a critical understanding of the application of theory and practice, to inform and advance clinical education.

MODULE STRUCTURE AND DELIVERY
The module is structured around five themed blocks which include a conference presentation block to facilitate the knowledge and skills to support leadership and supervision of others in an educational role. Both asynchronous and synchronous methods of teaching will be used. In addition, students will be required to design and undertake a work-based education improvement project which forms the basis of the formative and summative assessments within this module. All students will be allocated a project supervisor from the module team to support project development. Supervision time will also be available on synchronous teaching days for students to meet and discuss their project with their project supervisor. As part of this process, students will be required to complete a learning contract using the template provided (Appendix III). Supervisors will provide feedback on drafts and sign off the final version of the learning contract. **The agreed and signed off version will need to be discussed and appended within the summative written submission.**

The themed blocks within this module are as follows:

Block 1: Curriculum Planning
After an introduction to the module with a focus on the learning outcomes and the student led work based project, students will explore requirements around curriculum planning, to include development of learning outcomes, selection of teaching methods, assessment, and evaluation of a curriculum. There will be an opportunity for students to meet up with project supervisors to begin planning their work-based project.

Block 2: Learning Facilitation
Students will have the opportunity to explore different ways to facilitate learning. Sessions will include PBL/EBL, critical incidents, team-based learning, using audience response systems and facilitating learning via distance learning and MOOCs. Students will be able to discuss remotely with their project supervisors if required. Learning contracts will need to be agreed and signed off by the deadline provided.

Block 3: Assessment and Evaluation
We will build on material covered in the previous module on this topic and appraise different approaches to assessment of learning and evaluation of the learning experience. Students will be provided opportunities to learn how to appraise different student assessment methods and facilitator evaluation methods in preparation for block 4.

Block 4: Presentation Skills
In this block, students will present an aspect of their work-based education improvement project to their peers and a course tutor as a formative assessment. They will receive written feedback from their peers and tutor on their presentation skills, in preparation for the summative presentation. This also gives students the opportunity to learn from their peers on a variety of learning activities and initiatives in the workplace as well as the opportunity to provide constructive feedback to others. Presentations will be delivered in an online synchronous session in groups led by a tutor. Further details will be provided within the module.

Block 5: Conference Day
Students will be required to upload a video recording of a presentation on their work-based project as part of their summative assessment. They will receive a mark and written feedback on their presentation skills from their tutor. This mark will contribute to 10% of the module mark.

Students will be encouraged to read widely around topics of interest. Supervision time will also be available on each timetabled synchronous teaching day for students to plan and discuss their work-based education improvement project with their project supervisor. Asynchronous teaching will include opportunities for discussions with peers and module tutors as well as self-directed reading and suggestions for workplace-based learning and reflection.

MODULE OUTCOMES
By the end of the module (with reference to UKPSF outcomes) the students will be able to:

- Discuss and apply their knowledge and skills in supporting others who have an adult education role.
- Consider and implement assessment and the provision of feedback to others who have an education role, *which are appropriate to their workplace and role as a leader in education.*
- Engage with continuing professional development in their professional discipline and their pedagogy, incorporating research, scholarship and evaluation of professional practice.

- Research and appraise relevant subject material and educational theory in educational practice.
- Apply and critique methods of teaching, learning and assessment appropriate to their workplace and role as a leader in education.
- Use and discuss the value of a range of learning technologies.
- Implement and review methods for evaluating the effectiveness of teaching and appreciate how session/course/curriculum evaluation informs future planning and delivery
- Consider the implications of quality assurance and quality enhancement or academic and professional practice with a particular focus on teaching.
- Demonstrate and reflect upon the following professional values ;
    - Respect individual learners and diverse learning communities
    - Promote participation in higher education and equality of opportunity for learners
    - Use evidence-informed approaches and the outcomes from research, scholarship and continuing professional development
    - Acknowledge the wider context in which higher education operates recognising the implications for professional practice.

MODULE ASSESSMENT
The module is assessed in two parts:

<u>Part 1</u>
An evaluation of a work-based education improvement project relating to an aspect of leadership in teaching, learning and assessment (4,000 words). You will be allocated a project supervisor from the module team to support your project development.

Contributing 90% of the module mark.

The student will be required to:

1. Plan their project using a learning contract, to be referenced and appended within the project report.
2. Briefly describe and provide a critically reasoned defence for the focus for their work-based project.
3. Demonstrate a systematic approach to, planning, implementing and evaluating the chosen project. (If an evaluation is not possible within the time frame of the project, a plan for evaluation must be included).
4. Provide a critical appraisal of relevant evidence from a range of perspectives (including current research, PSRB, policy and educational theory).
5. Present clear evidence of insights and understandings gained or altered through engagement with the planning and delivery of the work based project.
6. Outline recommendations for their future learning, future practice and organisational development.

Evidence of the achievement of the learning, such as excerpts from reflective logs, documents produced to facilitate student's learning, selected highlights from student evaluations of their learning experiences, etc. should be appended to the project report.

Please ensure you remove all names, including your own, and work locations on all documentation so that your work is anonymised and you do not breech confidentiality.

Submission date: May (refer to e-vision for date)
Marking by module team (project supervisor) and moderation usually by the module coordinator.

Part 2
Students will be required to upload a 15-minute video presentation (see block 5) on an aspect of their work-based project which they consider to be relevant and of interest to their peers on the module.

Contributing 10% of the module mark.

Arrangements for the video presentations:
Presentations will be marked by their project supervisors and subject to moderation by a member of the module team; these will be made available to the external examiner who may view a sample of the presentations.

Both components of the assessment must be passed (over 50%) in order to pass the module.

Presentation Date: May (refer to e-vision for date)

Professional Recognition
The PGCertClinEd modules have been accredited for Fellowship of HEA (AFHEA) by Advance HE. Students completing this module will be encouraged to compile a portfolio of evidence against the UKPSF if they wish to apply for FHEA status. The application requires completion of a professional development log and a teaching observation report OR two testimonials from professional colleagues familiar with your educational activities. Please refer to section 2.13 for further details on the application process.

ARTICULATION OF MODULE LEARNING OUTCOMES WITH UK PSF (2011)

Module Learning Outcomes<br><br>At the end of the module the student will be able to:	UK PSF Areas of Activity, Core Knowledge and Professional Values (2011)
Discuss and apply their knowledge and skills in supporting others who have an adult education role.	Areas of Activity: 1, 2, 4<br><br>Core Knowledge: 1, 2, 3, 4<br><br>Professional Values: 1, 2, 3, 4
Consider and implement assessment and the provision of feedback to others who have an education role, which are appropriate to their workplace and role as a leader in education.	Areas of Activity: 3, 5,<br><br>Core Knowledge: 1, 2, 3, 4, 5, 6<br><br>Professional Values: 1, 3
Engage with continuing professional development in their professional discipline and their pedagogy, incorporating research, scholarship and evaluation of professional practice.	Areas of Activity: 5<br><br>Core Knowledge: 1, 2, 3, 4, 5, 6<br><br>Professional Values: 3, 4

Research and appraise relevant subject material and educational theory in educational practice.	Areas of Activity: 1,<br><br>Core Knowledge: 1, 2, 3, 4, 5<br><br>Professional Values: 1, 3, 4
Apply and critique methods of teaching, learning and assessment appropriate to their workplace and role as a leader in education.	Areas of Activity: 1, 4, 5<br><br>Core Knowledge: 1, 2, 3, 4, 5, 6<br><br>Professional Values: 1, 2, 3, 4
Use and discuss the value of a range of learning technologies.	Areas of Activity: 1, 2, 3, 4<br><br>Core Knowledge: 1, 2, 3, 4, 5<br><br>Professional Values: 1, 3
Implement and review methods for evaluating the effectiveness of teaching and appreciate how session/course/curriculum evaluation informs future planning and delivery	Areas of Activity: 1, 2,5<br><br>Core Knowledge: 1, 2, 3, 4, 5, 6<br><br>Professional Values: 1, 2, 3, 4
Consider the implications of quality assurance and quality enhancement on academic and professional practice with a particular focus on teaching.	Areas of Activity: 5<br><br>Core Knowledge: 1, 2, 3, 4, 5, 6<br><br>Professional Values: 1, 2
Demonstrate and reflect upon the following professional values:<br><br>- Respect individual learners and diverse learning communities<br>- Promote participation in higher education and equality of opportunity for learners<br>- Use evidence-informed approaches and the outcomes from research, scholarship and continuing professional development<br>- Acknowledge the wider context in which higher education operates recognising the implications for professional practice.	Areas of Activity: 1, 2, 3, 4, 5<br><br>Core Knowledge: 1, 2, 3, 4, 5, 6<br><br>Professional Values: 1, 2, 3, 4.

2.6.2 Module Descriptors for PGCert – Primary Care Tutor Mode

Students opting to study for the award of a Postgraduate Certificate in Clinical Education must successfully complete COMPULSORY module one (20 credits) and COMPULSORY module two (40 credit module). Successful completion of the PGCert modules will contribute to achievement of HEA professional recognition status at D1/D2 of UKPSF, 2011

MED-7053Y PRIMARY CARE TUTOR TRAINING MODULE 1 - CLINICAL SUPERVISOR (Compulsory; 20 credits). This module will not run in 20-21.

MODULE OVERVIEW

This module will introduce learners to basic principles of underpinning educational theory and practice required to enhance their clinical education role, particularly as a clinical supervisor, mentor, and practice educator. This module is specifically tailored to meet the needs for flexibility among primary care tutors who teach UEA medical students on placement in general practice. This introduction will form the foundations upon which to build further knowledge in module two.

This primary care tutor mode of the PGCertClinEd acknowledges the uniqueness of learning in clinical and practice settings and includes formal direct observation of teaching. This recognises the importance of the role of the educator in facilitating learning which occurs in the context of client care. Learners will be required to demonstrate the link between educational theory and their evolving development as an educator.

The overall aim of the module is to enable learners to acquire a body of knowledge and principles, through both face-to-face sessions and observation and reflection on real teaching, to facilitate teaching, learning and assessment in practice-based settings. This will enable them to work effectively with their students, who will become the workforce of the future. Ultimately this will improve services.

MODULE STRUCTURE/DELIVERY

The module is structured around three themes and delivered over three days, each of which is run as a full day workshop.

The three days/workshops are entitled:

- Self-development in clinical education
- The learning environment in clinical education
- Assessment and evaluation in clinical education

Each day will have associated self-directed and online activities, in the form of pre-reading and post-day work.

During the year, learners will also have a formative teaching observation carried out as part of a Quality Assessment Visit from the Professional Development Tutor (PDT). They will be required to write and submit their reflection on the teaching observed and the feedback provided, including the action plan, following the PDT visit.

Day 1: Self-development in clinical education
The day will cover presentation skills, teaching styles and methods for evaluating the effectiveness of teaching and learning strategies. Appraisal will also be covered.

Day 2: The learning environment in clinical education
This teaching day will explore how an effective learning environment can be established, how to establish and maintain an effective mentoring relationship and the key learning theories. Teaching examination skills and therapeutics will be discussed. Group teaching techniques will be covered.

Day 3: Assessment and evaluation in clinical education
This teaching day covers the principles of understanding learning outcomes and their role in assessment. It will also cover identifying and supporting the failing student, giving effective feedback and creating robust plans to manage the situation.

The learning and teaching strategies for the module will consist of lectures, group activities, opportunities for discussion and online learning. Each face-to-face teaching day will refer to educational theory, to stimulate reflection on the learners' own teaching practice and to encourage debate and further reading.

MODULE OUTCOMES
At the end of this module (with reference to the UKPSF) students will be able to:

- Design and plan learning activities appropriate to their workplace and role.
- Discuss and apply their knowledge and skills in teaching and/or supporting other adult learners).
- Consider and implement assessment and the provision of feedback to learners, which are appropriate to their workplace and role.
- Develop effective learning environments with support and guidance for other adult learners.
- Engage with continuing professional development in their professional discipline within a framework of reflection, incorporating research, scholarship and evaluation of professional practice.
- Research and appraise relevant subject material and educational theory in educational practice.
- Apply and critique methods of teaching, learning and assessment appropriate to their workplace and role as an educator.
- Explore own and other adult learners learning styles and preferences within their discipline.
- Implement and review methods for evaluating the effectiveness of teaching and appreciate how evaluation informs future planning and delivery.
- Demonstrate and reflect upon the following professional values:
    - Respect individual learners and diverse learning communities
    - Use evidence-informed approaches and the outcomes from research, scholarship and continuing professional development.

MODULE ASSESSMENT
The module is assessed in two parts:

Part 1 (formative assessment)
Quality Assessment Visit

The PDT will perform a visit with direct evaluation of the teaching in the workplace and provide feedback to the learner. Learners will be required to write and submit their reflections on the teaching observed and the feedback provided, including the action plan, following the PDT visit. This work will be reviewed formatively by the module lead and feedback will be provided.

Portfolio Requirements
Learners will be expected to compile a portfolio of evidence to demonstrate reflection and learning as an educator. The developing portfolio will be reviewed by the professional development tutor as part of the Quality Assessment visit. Content and learnings from the portfolio will also inform the summative, end of module essay.

Supporting material in the form of a portfolio will include:

1. Personal development plan and peer-to-peer appraisal (including details of educational PDP for the previous year and the outcome of these plans).
2. Report received from PDT after the Quality Assessment visit.
3. Written reflective piece, produced after the Quality Assessment visit.
4. Written feedback from students (Including that received directly after teaching and anonymised feedback received later).
5. A record of reflective discussion with the PDT on this feedback
6. Daily reflection on teaching day with relevant feedback from the PDT.
7. Reflection on GP tutor days that have been attended and demonstration of changes in understanding of learning and teaching following these sessions. It is expected that material from at least three GP tutor days over the past year will be reflected upon.

Part 2 (summative assessment)
Essay
Assignment title: Evaluation of your development as an educator and critical appraisal of educational theory and evidence.

A critical reflective essay (4000 words) on learning and developing as an educator. A reflective analysis of the learner’s personal development during the module.

This will draw on evidence collected for part 1.

You will be required to demonstrate:

1. Critical analysis of the factors that influence how learners integrate into clinical/educational practice settings and are able to meet defined learning outcomes
2. Critical evaluation of the necessary teaching and learning methods in clinical/educational practice settings in order to meet the needs of learners
3. Critical evaluation of the assessment process, including the provision of effective feedback to learners
4. Critical reflection of self and peer evaluation to facilitate continuing professional development in the role of the educator
5. Critical appraisal of relevant subject material and educational theory in educational practice.

Submission Date: July (refer to e-vision for date)
Marking by module team.

ARTICULATION OF MODULE LEARNING OUTCOMES WITH UK PSF (2011)

Module Learning Outcomes<br><br>At the end of the module the student will be able to:	UK PSF Areas of Activity, Core Knowledge and Professional Values (2011)
Design and plan learning activities appropriate to their workplace and role.	Areas of Activity: 1, 3, 4, 5,<br><br>Core Knowledge: 1, 3, 4,<br><br>Professional Values: 1, 2, 3, 4,
Discuss and apply their knowledge and skills in teaching and/or supporting other adult learners.	Areas of Activity: 2<br><br>Core Knowledge: 1, 2, 3,<br><br>Professional Values: 1, 2
Consider and implement assessment and the provision of feedback to learners, which are appropriate to their workplace and role.	Areas of Activity: 3, 4, 5,<br><br>Core Knowledge: 2, 3, 5, 6<br><br>Professional Values: 1, 3
Develop effective learning environments with support and guidance for other adult learners.	Areas of Activity: 2, 4,<br><br>Core Knowledge: 1, 2, 3, 4<br><br>Professional Values: 1, 2, 3, 4
Engage with continuing professional development in their professional discipline within a framework of reflection, incorporating research, scholarship and evaluation of professional practice.	Areas of Activity: 5<br><br>Core Knowledge: 1, 5, 6<br><br>Professional Values: 3, 4
Research and appraise relevant subject material and educational theory in educational practice.	Areas of Activity: 5<br><br>Core Knowledge: 5, 6<br><br>Professional Values: 3, 4
Apply and critique methods of teaching, learning and assessment appropriate to their workplace and role as an educator.	Areas of activity: 2, 5<br><br>Core Knowledge: 1, 2, 3,5 ,6<br><br>Professional Values: 1,3, 4
Explore own and other adult learners learning styles and preferences within their discipline.	Areas of Activity: 5<br><br>Core Knowledge: 2, 3,<br><br>Professional Values: 1, 3
Implement and review methods for evaluating the effectiveness of teaching and appreciate how evaluation informs future planning and delivery.	Areas of Activity: 2, 5<br><br>Core Knowledge: 5<br><br>Professional Values: 3

Demonstrate and reflect upon the following professional values;<br><br>• respect individual learners and diverse learning communities<br>• use evidence - informed approaches and the outcomes from research, scholarship and continuing professional development	Areas of Activity: 2, 5<br><br>Core Knowledge: 2, 3,<br><br>Professional Values: 1, 2, 3, 4

MED-7054Y Part A PRIMARY CARE TUTOR MODE MODULE 2 – EDUCATIONAL SUPERVISOR
(compulsory; one year part-time; 20 credits)

MED-7055Y Part B PRIMARY CARE TUTOR MODE MODULE 2 – EDUCATIONAL SUPERVISOR
(compulsory; one year part-time; 20 credits). This module will not run in 20-21.
Total = two years part time, 40 credits

MODULE OVERVIEW
This module will further develop the learners' knowledge of educational theory to encourage evidence-based practice within the clinical education role, particularly as a clinical supervisor, mentor, and practice educator. This will build on the basic foundations provided in module one when developing as a clinical supervisor. It will also develop the applicant's ability to take a leadership role within the workplace. It is specifically tailored to meet the needs of GP Tutors who wish to develop their skills to progress to a role in educational supervision and leadership. This module will also be relevant to clinicians working in secondary care. It is expected that the learner will develop a more extensive and deeper knowledge of educational theory, (including further knowledge of recommended texts) during this module. Learners will demonstrate the development and application of their new knowledge, both in observed practice as well as in their reflection and final essay.

MODULE STRUCTURE AND DELIVERY
The module is structured around four themes that will facilitate the knowledge and skills to support adult learners. These themes will be addressed through six teaching days delivered over two years. Each teaching day will be run as a full day workshop.

The four major themes are:

- Self-development in clinical education
- The learning environment in clinical education
- Assessment and evaluation in clinical education
- Interprofessional learning and team working

Each theme will be developed with spiral learning, based on previous teaching in module one. Content will be tailored to allow teaching and learning activities to match the extended learning needs for module two. Each day will have associated self-directed and online activities, in the form of pre-reading and post-day work.

During the year learners will also have a Quality Assessment Visit from the PDT. They will be required to write and submit a reflective piece on the quality assessment.

The learning and teaching strategies for the module will consist of lectures, group activities, opportunities for discussion and online learning. Each face-to-face teaching day will refer to educational theory, to stimulate reflection on the learners' own teaching practice and to encourage debate and further reading.

MODULE OUTCOMES
By the end of the module (with reference to UKPSF outcomes) the students will be able to:

- Discuss and apply their knowledge and skills in supporting others who have an adult education role.
- Consider and implement assessment and the provision of feedback to others who have an education role, *which are appropriate to their workplace and role as a leader in education.*
- Engage with continuing professional development in their professional discipline and their pedagogy, incorporating research, scholarship and evaluation of professional practice.
- Research and appraise relevant subject material and educational theory in educational practice.
- Apply and critique methods of teaching, learning and assessment appropriate to their workplace and role as a leader in education.
- Use and discuss the value of a range of learning technologies.
- Implement and review methods for evaluating the effectiveness of teaching and appreciate how session/course/curriculum evaluation informs future planning and delivery.
- Consider the implications of quality assurance and quality enhancement or academic and professional practice with a particular focus on teaching.
- Demonstrate and reflect upon the following professional values ;
    - Respect individual learners and diverse learning communities
    - Promote participation in higher education and equality of opportunity for learners
    - Use evidence-informed approaches and the outcomes from research, scholarship and continuing professional development
    - Acknowledge the wider context in which higher education operates recognising the implications for professional practice.

MODULE ASSESSMENT
The module will be assessed in two parts:

<u>Part 1 (formative assessment)</u>
Quality Assessment Visit
The PDT will perform a visit with direct evaluation of the teaching in the workplace. Written reports provided from this direct observation will be used by the learner to write a reflective piece. This work will be submitted and reviewed formatively by the module lead.

Portfolio Requirements
Learners will be expected to compile a portfolio of evidence to demonstrate reflection and learning as an educator. The developing portfolio will be reviewed by the PDT as part of the Quality Assessment Visit. Content and learnings from the portfolio will also inform the essay. The portfolio should demonstrate with evidence the learners' progression as an educator.
Supporting material in the form of a portfolio will include:

1. Personal development plan (PDP) and peer-to-peer appraisal (including details of educational PDP for the previous year and the outcome of these plans).
2. Report received from PDT after the Quality Assessment visit.

3. Written reflective piece, demonstrating educational progression during this module and corresponding to the PDP of the applicant. This will be produced after the Quality Assessment Visit.
4. Written feedback from students (Including that received directly after teaching and anonymised feedback received later).
5. A record of reflective discussion with the PDT on this feedback.
6. Daily reflection on teaching day, with relevant feedback from the PDT.
7. Reflection on GP tutor days that have been attended and demonstration of changes in understanding of learning and teaching following these sessions. It is expected that material from at least three GP tutor days over the past year will be reflected upon.
8. An agreed learning contract to carry out a work-based education improvement project relating to an aspect of leadership in teaching, learning and assessment. You will be allocated a project supervisor for this purpose. Drafts for feedback can be submitted ahead of this date and feedback will be provided for further development as required.

Part 2 (summative assessment)

An evaluation of a work-based education improvement project relating to an aspect of leadership in teaching, learning and assessment (4,000 words).

The student will be required to:

1. Plan their project using a learning contract, to be referenced and appended within the project report.
2. Briefly describe and provide a critically reasoned defence for the focus for their work-based project.
3. Demonstrate a systematic approach to, planning, implementing and evaluating the chosen project. (If an evaluation is not possible within the time frame of the project, a plan for evaluation must be included).
4. Provide a critical appraisal of relevant evidence from a range of perspectives (including current research, PSRB, policy and educational theory).
5. Present clear evidence of insights and understandings gained or altered through engagement with the planning and delivery of the work-based project.
6. Outline recommendations for their future learning, future practice and organisational development.

Evidence of the achievement of the learning, such as excerpts from reflective logs, documents produced to facilitate student's learning, selected highlights from student evaluations of their learning experiences, etc. should be appended to the project report.

Please ensure you remove all names, including your own, and work locations on all documentation so that your work is anonymised and you do not breech confidentiality.

Submission Date: July (refer to e-vision for date)
Marking by module team.

ARTICULATION OF MODULE LEARNING OUTCOMES WITH UK PSF (2011)

Module Learning Outcomes<br><br>At the end of the module the student will be able to:	UK PSF Areas of Activity, Core Knowledge and Professional Values (2011)
Discuss and apply their knowledge and skills in supporting others who have an adult education role.	Areas of Activity: 1, 2, 4<br><br>Core Knowledge: 1, 2, 3, 4<br><br>Professional Values: 1, 2, 3, 4
Consider and implement assessment and the provision of feedback to others who have an education role, which are appropriate to their workplace and role as a leader in education.	Areas of Activity: 3, 5,<br><br>Core Knowledge: 1, 2, 3, 4, 5, 6<br><br>Professional Values: 1, 3
Engage with continuing professional development in their professional discipline and their pedagogy, incorporating research, scholarship and evaluation of professional practice.	Areas of Activity: 5<br><br>Core Knowledge: 1, 2, 3, 4, 5, 6<br><br>Professional Values: 3, 4
Research and appraise relevant subject material and educational theory in educational practice.	Areas of Activity: 1,<br><br>Core Knowledge: 1, 2, 3, 4, 5<br><br>Professional Values: 1, 3, 4
Apply and critique methods of teaching, learning and assessment appropriate to their workplace and role as a leader in education.	Areas of Activity: 1, 4, 5<br><br>Core Knowledge: 1, 2, 3, 4, 5, 6<br><br>Professional Values: 1, 2, 3, 4
Use and discuss the value of a range of learning technologies.	Areas of Activity: 1, 2, 3, 4<br><br>Core Knowledge: 1, 2, 3, 4, 5<br><br>Professional Values: 1, 3
Implement and review methods for evaluating the effectiveness of teaching and appreciate how session/course/curriculum evaluation informs future planning and delivery	Areas of Activity: 1, 2,5<br><br>Core Knowledge: 1, 2, 3, 4, 5, 6<br><br>Professional Values: 1, 2, 3, 4
Consider the implications of quality assurance and quality enhancement on academic and professional practice with a particular focus on teaching.	Areas of Activity: 5<br><br>Core Knowledge: 1, 2, 3, 4, 5, 6<br><br>Professional Values: 1, 2
Demonstrate and reflect upon the following professional values:<br><br>- Respect individual learners and diverse learning communities<br>- Promote participation in higher education and equality of opportunity for learners	Areas of Activity: 1, 2, 3, 4, 5<br><br>Core Knowledge: 1, 2, 3, 4, 5, 6<br><br>Professional Values: 1, 2, 3, 4.

- Use evidence-informed approaches and the outcomes from research, scholarship and continuing professional development<br>- Acknowledge the wider context in which higher education operates recognising the implications for professional practice.	

2.6.3 Module Descriptors for PGDip and Masters Modules

Students opting to study for the award of a Postgraduate Diploma in Clinical Education must successfully complete the previous TWO COMPULSORY 20 and 40 Credit modules plus ONE FURTHER COMPULSORY module, one CORE module and choose one CORE of two 20 credit taught OPTIONS. Teaching for these modules will be delivered remotely.

MED-7020F LEADING CONTEMPORARY CLINICAL EDUCATION (compulsory; 20 credits)

MODULE OVERVIEW

This module is geared towards practising healthcare professionals who are interested in contemporary approaches to clinical education. It will provide an overview of current pedagogical methods and leadership for curricular change in a constantly changing world. It will also help inform, develop and prepare health professionals in the use of new technology including social media to enhance their teaching and training activities delivered in the context of continuing professional development and lifelong learning for a diverse workforce.

MODULE STRUCTURE AND DELIVERY

This module will be delivered using synchronous and asynchronous methods over two themed blocks delivered via Blackboard, the UEA's virtual learning environment. Students will be expected to engage fully with the course material and participants through virtual discussion groups throughout the module. Optional Blackboard training sessions can be arranged for students who are not familiar with the use of Blackboard.

The two themed blocks consist of a mix of plenary sessions and group work delivered synchronously/ asynchronously. The themes are:

- Contemporary Pedagogy in Clinical Education
- Technology Enhanced Learning in Clinical Education

Leading Change in Clinical Education will also be addressed throughout the module.

MODULE OUTCOMES

By the end of this module, students will be able to:

- Evaluate contemporary subject and pedagogic research and/or scholarship activities as part of evidence-based teaching and learning.
- Critically analyse current trends in methods of teaching and facilitating learning in clinical education.
- Demonstrate the use of innovative technologies to enhance learning, and critically evaluate the strengths and weaknesses of each.

- Demonstrate effective educational leadership capabilities applied to a range of knowledge and educational interventions and programmes.
- Design an educational activity for a group of healthcare professionals using justified evidence-based methodology, and an evaluation plan.

MODULE ASSESSMENT

This will consist of (a) a formative mid-module essay; (b) two summative online tasks linked to Blackboard discussion boards, raising questions linked to curricular objectives about evaluating research and trends in education (c) a summative essay completed at the end of the module.

The **formative assessment** will consist of a 1,000 word report developing a lesson plan for a teaching/ training session using one form of technology appropriate for the session, based on a reasoned discussion including a consideration of its strengths and weaknesses.

Two online tasks will be summatively assessed after delivery of the two online blocks. The summative assessment weighting for each task will be 15 % (30 % in total).

The **summative assessment** will also include a 2,500 word essay developing and justifying a lesson plan incorporating the use of at least one form of educational technology for the delivery of a teaching/ training session. Students are expected to expand on the lesson plan developed in the formative assessment using feedback provided for the purpose of this summative assessment. The justification should include a critical appraisal of published evidence to support choice of technology including a consideration of its strengths and weaknesses; plan for delivery of teaching/training, resources required, method of student assessment in terms of achievement of the session learning outcomes, and criteria for evaluation of the successful delivery of the planned session. The summative assessment weighting for this task will be 70 % of overall marks.

Submission Date: July (refer to e-vision for date)
Marking by module team.

ARTICULATION OF MODULE WITH UK PSF

Module Outcomes	UK PSF Areas of Activity, Compulsory Knowledge and Professional Values
Evaluate contemporary subject and pedagogic research and/or scholarship activities as part of evidence-based teaching and learning.	Areas of Activity: A 4, 5<br>Core Knowledge: K 1, 2, 3, 4, 5<br>Professional Values: V 3, 4
Critically analyse current trends in methods for teaching and facilitating learning in clinical education.	Areas of Activity: A 1, 2, 3, 4<br>Core Knowledge: K 1, 2, 3, 4, 5<br>Professional Values: V 3, 4
Demonstrate the use of innovative technologies to enhance learning, and critically evaluate the strengths and weaknesses of each.	Areas of Activity: A 1, 2, 3, 4<br>Core Knowledge: K 1, 2, 3, 4, 5<br>Professional Values: V 1, 2, 3, 4
Demonstrate effective educational leadership capabilities applied to a range of knowledge and educational interventions and programmes.	Areas of Activity: A 4, 5<br>Core Knowledge: K 2, 3, 4, 5, 6<br>Professional Values: V 1, 2, 3, 4

Design an educational activity for a group of healthcare professionals using justified evidence-based methodology, and an evaluation plan.	Areas of Activity: A 1, 2, 3, 4, 5<br>Core Knowledge: K 1, 2, 3, 4, 5, 6<br>Professional Values: V 1, 3, 4

MED-7021D INTRODUCTION TO RESEARCH METHODS (core; 20 credits)

This module aims to introduce students to both qualitative and quantitative research design and methods issues; such that students are able to critique research reports and research-based peer reviewed journal articles. The module covers the following areas for both quantitative and qualitative research paradigms: Introduction to Enquiry, Identifying Research Questions, Data Collection Designs, Approaches to Data Analysis, Issues of Presentation. Understandings of the above issues enable students to become critical consumers of research, essential in their role as clinical supervisors and clinical educators.

MODULE OUTCOMES
Through this module students will:

- Develop skills and knowledge in evaluating research designs and published research that are relevant to an appropriate research area;
- Gain experience as an actively participating research group member in the verbal and written communication of critical appraisal of research designs and publications;
- Know the rationale for, and be able to evaluate the use of, surveys, randomised controlled trials and qualitative studies;
- Appreciate the principles under-pinning inferential statistical methods and be able to interpret, and evaluate the use of, hypothesis tests and confidence intervals in a variety of contexts;
- Appreciate the principles underpinning diverse qualitative design approaches and be able to interpret and evaluate their use in addressing; and
- Understand the potential sources of error that may compromise the internal and external validity or trustworthiness of a study.

MODULE ASSESSMENT
- Online course exam of quantitative research (50% of assessment, 1.5 hours)
- Online course exam of qualitative research (50% of assessment, 1.5 hours)

For an overall pass mark, both course exams must be at least 50%.

Choose 1 of the following 3 CORE research modules:

MED-7013E FURTHER QUANTITATIVE RESEARCH METHODS (*NB. for students who wish to go on and complete a Masters rather than a PG Diploma, this module or Further Qualitative methods module OR Systematic Reviews module will become CORE; 20 credits*)

This module aims to build on the coverage of quantitative methods and critical appraisal skills in 'Introduction to Research Methods'. The module will enable students to gain further knowledge of the conduct of quantitative studies and equip them with practical skills in planning and designing quantitative research studies, data collection, quantitative analysis and interpretation. The module is delivered through ten x four-hour sessions. Six of the sessions involve lectures and group discussions. The other four sessions are based in a computer laboratory and involve tuition and structured

practical and private work intended to help the student develop strategies to organise and analyse quantitative research data.

MODULE OUTCOMES
At the end of this module the student will have:

- Drawn up an initial plan for a research project – identify a topic, generate a research question and choose appropriate methods and design to investigate it.
- Demonstrated that they have gained further understand approaches to reviewing and summarising a body of literature and the problems in doing this.
- Evaluated methods of outcome measurement.
- Demonstrated that they have added further depth to their understanding of statistical methods progressing beyond understanding of the basics of descriptive and inferential statistics, to an understanding of the application of non-parametric tests, chi-squared and related tests, and correlation coefficients.
- Applied the statistical knowledge gained on MED-7021D/E and within this module to a dataset, to meaningfully present and analyse the data using statistical methods.

MODULE ASSESSMENT
- Summative data analysis exercise - data manipulation, summarising and analysing a dataset, and producing a written report on the findings (max. 2000 words).
- Design a research protocol (2000 word assignment).
- Formative data analysis exercise

MED-7014E FURTHER QUALITATIVE RESEARCH METHODS

(*NB. for students who wish to go on and complete a Masters rather than a PG Diploma, this module or Further Quantitative methods module or Systematic Reviews module will become CORE; 20 credits***)**

This module aims to build on the coverage of qualitative methods and critical appraisal skills in 'Introduction to Research Methods'. The module will enable students to gain further knowledge of the conduct of qualitative studies and equip them with practical skills in planning and designing qualitative research studies, data collection, qualitative analysis and interpretation.

The module will comprise 32 hours of contact. This is arranged as eight study days where you will be required to attend University for taught theory and group activities.

The module will be delivered through a 'blended' learning approach as:

- Narrated PowerPoint lectures
- Group and student-led presentations
- Structured group activities as formative course work
- Web-based activities
- Individual and group tutorials

The delivery of the core content of this module will reflect a thematic approach by building, session by session, your knowledge base in a systematic and structured way. The group task, as the formative elements within this module, are structured in such a way as to support you to apply new knowledge, prepare for your summative assignments and engage in professional dialogue within a supportive learning environment.

MODULE OUTCOMES
At the end of this module the student will:

- Develop skills in planning and conducting a qualitative study that are relevant to an appropriately selected research question.
- Demonstrate a critical awareness of the development of these skills in the context of group presentations, discussions and peer review.
- Show an awareness of ethical issues arising in the course of qualitative health and social research.
- Gain experience, as an actively participating research group member, in the verbal communication of a qualitative research proposal and qualitative project work.
- Develop the knowledge and skills necessary to individually produce a written report of group project work (including critical reflections and some data analysis) and a well-planned research proposal.

MODULE ASSESSMENT
Written research proposal (4,000 words).

HSC-7057E SYSTEMATIC REVIEWS AND RESEARCH SYNTHESIS (*NB. for students who wish to go on and complete a Masters rather than a PG Diploma, this module or Further Quantitative methods module or Systematic Reviews module will become CORE; 20 credits*)

MODULE DESCRIPTION
Findings from systematic reviews have been increasingly used by health policy makers, clinicians and patients for making decisions. A systematic review of available evidence is also often required for developing new research, and for interpreting findings from a primary study. The module will include the following contents:

- introduction, framing questions, inclusion/exclusion criteria
- sources of evidence and literature search strategy
- data extraction, and validity assessment
- synthesizing evidence from qualitative studies
- synthesizing evidence from quantitative studies
- quality of systematic reviews, and overview of reviews
- recent development in research synthesis methods
- systematic review protocol

Learning outcome: Provides students with the skills and understanding to appraise and interpret published systematic reviews, to develop a protocol and undertake a systematic review. By the end of the module, students will be able to:

- frame questions appropriate for a systematic review
- design an appropriate literature search strategy
- assess the relevance and quality of primary studies
- qualitatively and quantitatively synthesize data from primary studies
- appropriately interpret findings of a systematic review and meta-analysis
- understand common pitfalls in systematic reviews and meta-analysis
- become familiar with recent method research relevant to systematic reviews

Assessment: 100% Coursework

Students opting to study for the award of a Master of Clinical Education must successfully complete the previous Modules – totalling 20 Credits (including one of the Further Research modules) AND MUST successfully complete a 60-credit research dissertation on a topic related to teaching and/or learning.

HSC-7015X RESEARCH DISSERTATION

This module aims to allow students to illustrate the depth of knowledge that they have developed through the whole of their MClinEd and to give students practical experience in conducting research. All students are encouraged to consider their dissertation work for publication, either through the university or in refereed academic journals33-4. Research studies carried out by students registered for the MClinEd degree must focus on topics r that directly relate to teaching and/or learning for the clinical setting or clinical learning environments.

MODULE OUTCOMES

At the end of this module the student will have:

- Planned a research project.
- Critically evaluated the literature relating to their chosen topic.
- Demonstrated an understanding of the research methods appropriate to their topic.
- Presented a clear summary of the findings of their research.
- Critically analysed their findings and discussed the implications of their findings for theory and/or practice.

MODULE ASSESSMENT

Completion of a dissertation (15,000 words).

Further details about this module are provided in a separate dissertation handbook which will be made available to students via Blackboard.

2.7 Calendar of Modules Available within the Clinical Education Programme

The calendar below shows in which part of the academic year different modules via both the part time and fulltime routes over 2020-21:

PART TIME STUDY

**STANDARD/ ONLINE MODE**<br>**Part Time Year 1**<br>**(over 1 year)**		**PRIMARY CARE TUTOR MODE**<br>**Part Time Year 1**<br>**(over 3 years)**		
**Autumn Term**	HSC-7165D Enhancing Teaching, Learning and Assessment in Clinical Education	**Year 1 of study**	MED-7053Y MODULE 1 - Clinical Supervisor	*20 credits*
	AND		AND	
**Spring**<br>**AND**<br>**Summer**<br>**Term**	HSC-7166H Leading Teaching, Learning and Assessment in Clinical Education	**Year 2 of Study**<br>**Year 3 of Study**	MED-7054Y MODULE 2 - Educational Supervisor Part A<br>MED-7055Y MODULE 2 - Educational Supervisor Part B	*40 credits*
	Total - 60 Credits<br>*Eligible for Award of PGCertClinEd and Associate Fellowship/ Fellowship of HEA*			

Please note that from 2021, the online PG Certificate in Clinical Education will change to a January start date. Thus, from Jan 2021 onwards, module HSC-7165E Enhancing Teaching, Learning and Assessment in Clinical Education will run in the Spring Term and module HSC-7166X Leading Teaching, Learning and Assessment in Clinical Education will run over the Summer and Autumn terms. The face to face courses will continue to run from Sept to August each year.

Part Time Year 2		
**Autumn Term**	MED-7021D Introduction to Research Methods (CORE)	*20 credits*
**Spring Term**	Students must undertake one CORE module selected from:<br>MED-7013E Further Quantitative Research Methods<br>OR<br>MED-7014E Further Qualitative Research Methods<br>OR<br>HSC-7057E Systematic Reviews & Research Synthesis<br>***NB*** *Students studying for the award of MClinEd must have successfully completed either as one of their Spring Options before they can undertake the third year Research Dissertation module*	*20 credits*
**Summer Term**	MED-7020F Leading Contemporary Clinical Education (COMPULSORY)	*20 credits*
	Total - 120 credits<br>*Eligible for Award of PGDipClinEd*	

<table>
<tr><th colspan="3">Part Time Year 3</th></tr>
<tr><td>Autumn Term

Spring Term

Summer Term</td><td>↑
HSC-7015X Research Dissertation
Options:
• Systematic Literature Review
• Primary Research using Qualitative methods
• Primary Research using Quantitative methods
• Primary Research using Mixed Methods
• Secondary Analyses of Quantitative or Qualitative data
Topic directly related to teaching and/or learning
↓</td><td>60 credits</td></tr>
<tr><td></td><td colspan="2">Total - 180 credits
Eligible for Award of MClinEd</td></tr>
</table>

FULL TIME STUDY

<table>
<tr><th colspan="4">Full Time over One Year</th></tr>
<tr><td></td><td colspan="2">Taught Modules</td><td>Dissertation Module</td></tr>
<tr><td>Autumn Term</td><td>HSC-7165D Enhancing Teaching, Learning and Assessment in Clinical Education

(COMPULSORY - 20 credits)

AND

MED-7021D Introduction to Research Methods

(CORE - 20 credits)</td><td></td><td rowspan="4">NB Students studying for the award of MClinEd <u>must</u> have successfully completed either ‘Further Quantitative Research’

OR ‘Further Qualitative Research’

OR ‘Systematic Reviews & Research Synthesis’

in order to be permitted to register for the Research Dissertation module

HSC-7015X Research Dissertation

Topic directly related to Teaching and/ or Learning for the clinical setting or clinical learning environments.

(60 credits)</td></tr>
<tr><td>Spring Term</td><td rowspan="2">HSC-7166H Leading Teaching, Learning and Assessment in Clinical Education

(COMPULSORY – 40 credits)</td><td>MED-7013E Further Quantitative Research Methods

OR

MED-7014E Further Qualitative Research Methods

OR

HSC-7057E Systematic Reviews & Research Synthesis

(CORE 20 credits)</td></tr>
<tr><td>Summer Term</td><td>MED-7020F Leading Contemporary Clinical Education

(COMPULSORY – 20 credits)</td></tr>
<tr><td>July to Sept</td><td>No Taught modules</td><td>No Taught modules</td></tr>
<tr><td></td><td colspan="3">Total - 180 credits

Eligible for Award of MClinEd</td></tr>
</table>

2.8 Students Returning to MBBS Studies following MClinEd Completion

Students who have intercalated from their MBBS studies to undertake the MClinEd, and who will subsequently return to Year 5 of the MBBS programme, may face particular challenges and concerns regarding teaching and assessment on the MB/BS programme. The Head of Norwich Medical School and the Director of the MB/BS programme have undertaken to manage the teaching and assessment of these students by personnel who may have been the students' peers on the MClinEd. Specifically, Year 5 MBBS students will not be assessed by any MBBS members of staff who have previously been their peers/colleagues on the MClinEd. Wherever possible, for small group teaching, Year 5 MBBS students will be allocated to groups taught by staff who have not been their peers on the MClinEd. During large group teaching sessions, it may not be possible to avoid contact between students and staff who have been colleagues on the Masters programme. In these situations, staff will be sensitive to needs of Year 5 students and will secure appropriate guidance and support from HOS and Director in managing this issue. Students will not be assessed by staff members who have been their peers on the MClinEd.

2.9 Access to Work Based Opportunities for Clinical Education Programme studies

For work-based project and research elements of the programme, it may be necessary for some students to negotiate access to HSC or MED teaching and HSC or MED students and teaching staff. This access should be sought via the relevant Head of School:

MED – Professor William Fraser (PA email: med.hospa@uea.ac.uk or phone: 01603 593959)

HSC – Professor Sally Hardy (PA email: megan.hogg@uea.ac.uk or phone: 01603 591724)

MClinEd students are advised that on the whole, MBBS students based in MED are already a heavily evaluated group of students and wherever possible, work-based projects and research undertaken for the MClinEd should try to find alternative groups of students upon which to focus.

2.10 Pastoral Care and Academic Advice for Students on the Clinical Education Programme

All students will be allocated a personal advisor. This member of faculty should be your first point of contact should you have any problems that interfere with your engagement with your studies. They may also be able to advise and support you with general academic matters such as academic writing and referencing. On the Clinical Education programme, we are keen for students to experience good mentoring and supervision in order that they may draw upon these experiences in their support of other people's learning. To this end, the programme team have agreed to make themselves available to their personal advisees for a minimum of two supervisions per year, to review the student's progress. Students are responsible for booking these appointments with their advisor at points during the academic year that they deem to be most suitable for their needs. They are also responsible for preparing for these tutorials, for example alerting the advisor in advance of the issues that they particularly wish to discuss and having taken time to reflect on their progress prior to the meeting. In addition, students may seek one to one supervision from members of module teams regarding module work and their personal/professional development through any given module. This will include an opportunity to share and discuss the mapping of the portfolio of evidence to the UKPSF for those seeking HEA Associate Fellowship or Fellowship.

2.11 Clinical Education Programme Module Organisers

HSC-7065D	Enhancing Teaching, Learning and Assessment in Clinical Education	Nicki Young	Nicki.young@uea.ac.uk
HSC-7165D	Enhancing Teaching, Learning and Assessment in Clinical Education	Veena Rodrigues	v.rodrigues@uea.ac.uk
HSC-7066H	Leading Teaching, Learning and Assessment in Clinical Education	Emma Parker-Jaggard	E.Parker@uea.ac.uk
HSC-7166H	Leading Teaching, Learning and Assessment in Clinical Education	Emma Parker-Jaggard	E.Parker@uea.ac.uk
MED-7021D	Introduction to Research Methods	Sarah Hanson & Allan Clark	S.Hanson@uea.ac.uk Allan.clark@uea.ac.uk
MED-7020F	Leading Contemporary Clinical Education	Barbara Jennings	b.jennings@uea.ac.uk
MED-7013E	Further Quantitative Research Methods	Jane Skinner	Jane.skinner@uea.ac.uk
MED-7014E	Further Qualitative Research Methods	Joanna Semlyen	j.semlyen@uea.ac.uk
HSC-7057E	Systematic Review & Research Synthesis	Jane Cross	j.cross@uea.ac.uk
HSC-7015X	Research Dissertation	Diane Bunn	D.Bunn@uea.ac.uk

2.12 New Appointments of FMH Lecturers

<table>
<tr><td colspan="3" align="center">FMH Lecturer Appointment

Line Manager (on behalf of Head of School) to identify at interview and appointment

↙ ↘</td></tr>
<tr><td>FMH</td><td></td><td>EDU</td></tr>
<tr><td>ClinEd Route</td><td></td><td>HEP Route</td></tr>
<tr><td>PGCert Clin Ed</td><td></td><td>PGCert HEP</td></tr>
<tr><td colspan="3" align="center">Line manager to notify Human Resources about decision</td></tr>
<tr><td colspan="3" align="center">Appointment Letter sent</td></tr>
<tr><td colspan="3" align="center">Staff application to Admissions as appropriate

↙ ↘</td></tr>
<tr><td>FMH Admissions

hsc.pgt.admiss@uea.ac.uk

Application flagged as Staff Member</td><td></td><td>EDU PGT Admissions</td></tr>
<tr><td colspan="3" align="center">Registration

Year 1 of appointment</td></tr>
<tr><td>Complete PGCert ClinEd (12 months)</td><td></td><td>Complete PGCert HEP (24 months)</td></tr>
<tr><td colspan="3" align="center">Apply via Course Directors for recognition as FHEA</td></tr>
</table>

2.13 Professional Recognition (Associate Fellowship/ Fellowship of HEA)
The PGCertClinEd modules/course are accredited by Advance HE for professional recognition at D1 (Associate Fellowship)/D2 (Fellowship). This section provides further information on this process and includes all documentation you will need for this purpose.

2.13.1 Frequently asked questions

Am I eligible to apply for HEA professional recognition?
Students who have successfully completed the following modules will be eligible for recognition by the HEA at **D1 (associate fellow),** once they have submitted and passed the professional development log reflecting on their portfolio of evidence and demonstrating achievement of **the appropriate dimensions/D1 descriptor** of the UKPSF, along with a teaching observation report or two testimonials from colleagues familiar with their educational activities.

Standard HSC-7065D OR Online Mode HSC-7165D/HSC-7165E (20 credit module)<br><br>*Enhancing teaching learning and Assessment in clinical education*	Primary Care Tutor Mode (20 credit module)<br><br>*MED-7053Y Module 1 - Clinical Supervisor*

Students who have successfully completed the PGCertClinEd which includes both of the following 2 modules will be eligible for recognition by Advance HE **at D2 level (fellow),** once they have submitted and passed the professional development log reflecting on their portfolio of evidence, including a teaching observation or two testimonials from colleagues familiar with their educational activities, and demonstrating achievement of **all dimensions/D2 descriptor** of the UKPSF.

PGCertClinEd - Standard HSC-7065D OR Online Mode HSC-7165D/ HSC-7165E (20 credit module)<br><br>*Enhancing teaching learning and Assessment in clinical education*<br><br>And<br><br>Standard HSC-7066H OR Online Mode HSC-7166H/ lHSC-7166X (40 credit module)<br><br>*Leading teaching learning and assessment in clinical education*	PGCertClinEd - Primary Care Tutor Mode (60 credits)<br><br>*MED-7053Y Module 1 - Clinical Supervisor*<br><br>*And*<br><br>*MED-7054Y Module 2 – Educational Supervisor Part A*<br><br>*MED-7055Y Module 2 – Educational Supervisor Part B*

<u>How do I present my evidence?</u>
You will be required to complete a **professional development log** (see section 2.13.2) reflecting upon your development as an educator and demonstrating how you meet the dimensions of the UKPSF at D1 or D2 level, collating evidence against the Areas of Activity, Core Knowledge, and Professional Values in your portfolio. Your portfolio can include evidence from previous educational roles/activities as well as that collected whilst studying on the course, including your teaching observation report/ testimonials and your reflections and any action plans from this.

The assignments within the course are designed to assess each module's learning outcomes. The course and module learning outcomes are mapped to the dimensions of the UKPSF and so the taught activities are aligned to the D1/D2 descriptors across the framework.

Your portfolio MUST include at least ONE completed teaching observation form commenting on the UKPSF dimensions of practice. You will need to make arrangements for doing this yourself. If you are unable to do this, you will be required to submit two **testimonials from professional colleagues** familiar with your educational activities in your workplace. Forms are provided in this handbook in section 2.13.3 and will be available to download from the course Blackboard site.

Your professional development log and teaching observation report or testimonials must be submitted through the course BlackBoard site. You do not need to submit your portfolio.

<u>Can I include evidence from my previous educator activities in my portfolio?</u>
Yes, you can do this. The evidence also needs to be mapped to the appropriate descriptors against the dimensions of the UKPSF in your professional development log so that it can be considered in making the professional recognition judgement.

<u>Who makes the professional recognition judgement about my portfolio of evidence?</u>
Two suitably trained reviewers from the MClinEd course team holding Senior Fellowship of HEA will assess your professional development log and action plan, your portfolio of evidence including the

teaching observation or testimonials using the professional recognition assessment form provided in this handbook in section 2.13.4.

If a student completing the 60-credit course is not able to demonstrate clear evidence against D2 descriptors across all dimensions, they will be offered the option of professional recognition at D1, or support to collate additional evidence for reassessment of the professional recognition judgement at D2. Detailed feedback will be provided in such cases to facilitate this process.

What happens next?
As a quality assurance mechanism (see section 2.13.5), a Senior Fellow of HEA, within UEA, but external to the course team, will review all submissions. Each year, a 10% sample of successful submissions and any unsuccessful submissions will also be reviewed by an external reviewer who is a Principal Fellow of HEA.

Following the exam board in November each year, the course director will contact all students who have submitted their portfolio of evidence intending to apply for professional recognition by HEA. If you have successfully met the requirements for professional recognition at D1/D2 level (see previous question), you will then be asked to provide your personal details required for submission to HEA.

The Course Director will upload names of students successful at D1/D2 to the HEA website. The HEA will contact you directly to arrange payment where appropriate, and to send you your certificate.

Do I need to pay?
HEA accreditation and subsequent fee-free professional recognition is a benefit to those employed within subscribing institutions. Those not employed by the University, or another HEA subscribing institution, will be liable for an additional fee for professional recognition on completion of the course. Please refer to the HEA website for the additional current fee due for professional recognition: https://www.heacademy.ac.uk/individuals/fellowship

What will happen if I am not successful in meeting the criteria for professional recognition?
You will be offered an opportunity to re-submit your portfolio. Guidance will be provided by a member of the course team including a review of your documentation and action plan. If you do not meet criteria for recognition at D2, you might be eligible for recognition at D1. Please note that you might require additional time to gain further experience to map as evidence for recognition at D2.

Will I be eligible for professional recognition by HEA if I complete the online mode of the PGCertClinEd?
Yes, completing the online PGCertClinEd modules (HSC-7165D and/or HSC-7166D) and successfully submitting your portfolio of evidence will provide all the eligibility requirements for AFHEA/FHEA status.

2.13.2 Professional Development Log

HEA Professional Recognition at D1 (Associate Fellowship) or D2 (Fellowship) level

Mapping of portfolio of evidence to demonstrate engagement with the UK Professional Standards Framework (2011)

<u>What you need to do</u>

Please reflect upon your development as an educator as you progress through the course. Consider your knowledge, activities, skills and practice in demonstrating how you meet the dimensions of the UK Professional Standards Framework (2011) at D1 or D2 level, collating evidence against the Areas of Activity, Core Knowledge, and Professional Values in your portfolio. Your portfolio can include evidence from previous educational roles/activities as well as that collected whilst studying on the course, including your teaching observation report/ testimonials and your reflections and any action plans from this.

At the end of your course, if you would like to apply for professional recognition from the Higher Education Academy for Associate Fellowship (D1) or Fellowship (D2), please complete this document, stating the items of evidence contained in your portfolio in relation to each dimension of the UKPSF.

Please submit a short reflective commentary referring to your evidence and evaluating your practice as an educator (D1 or D2 level) using the proforma below, including an action plan for your continuing professional development as an educator in your professional role. **You do not need to submit your portfolio**. Submission deadlines will be communicated to you via the Course site on Blackboard.

Dimensions of the UKPSF (2011)	Items of evidence in your portfolio	Reflective commentary on the evidence and how it aligns to the UKPSF
**Areas of Activity**		
A1.Design and plan learning activities and/or programmes of study		
A2.Teach and/or support student learning		

A3.Assess and give feedback to learners.		
A4.Develop effective learning environments and approaches to student support and guidance.		
A5.Engage in continuing professional development in subjects/disciplines and their pedagogy, incorporating research, scholarship and the evaluation of professional practices		
**Core Knowledge**		
K1. The subject material		
K2. Appropriate methods for teaching, learning and assessing in the subject area and at the level of the academic programme.		
K3. How students learn, both generally and within their subject/disciplinary area (s)		
K4. The use and value of appropriate learning technologies.		

K5. Methods for evaluating the effectiveness of teaching.		
K6. The implications of quality assurance and quality enhancement for academic and professional practice		
**Professional Values**		
V1. Respect individual learners and diverse learning communities		
V2. Promote participation in higher education and equality of opportunity for learners		
V3. Use evidence informed approaches and the outcomes from research, scholarship and continuing professional development.		
V4. Acknowledge the wider context in which higher education operates recognising the implications for professional practice.		

Continuing Professional Development Action Plan

Create an action plan for your continuing professional development as an educator based on your reflections in the previous section.

2.13.3 Observation of Teaching Form

Part 1: Pre-Observation Meeting

Date: ………………….

Observer: ………………………………………………………………………………………

Observee: ………………………………………………………………………………………

The pre-observation discussion should cover the following:

- How the Teaching session to be observed provides opportunities to observe the UKPSF Dimensions of Practice.
- The teaching approach to be adopted, anticipated student activities, approximate time plan for the session
- The context of the teaching (room, timetable, the status and history of the student group, any particular constraints or opportunities)
- How the observation is to be conducted (time of arrival and leaving, any focus to the observation, where observer will sit, etc.)
- The time and place of the observation and feedback
- The content and its place within the curriculum of the unit and the programme of study
- The way in which the students will be informed and incorporated into the observation
- Tutor's expectations/objectives for the teaching session.
- Any particular aspects that the tutor wishes to have observed (e.g., design, structure, interaction with students, verbal and non-verbal communication)
- Any particular concerns that either the observer or the observed might have about undertaking the observation

Please continue on a separate sheet if required

Part 2: Observation Notes

Date: ………………….

Observer: ……………………………………………………………………………………

Observee: ……………………………………………………………………………………

The Session being observed

Subject	Course Unit / Module	Title of Topic
Length of Session	Observation Time	Type of activity (e.g. lecture, seminar, tutorial, online learning)
Level/Year	Mode (FT/PT/Online)	Number of students at session

The sections below identify the Dimensions of the UKPSF linked to the observation which need to be met.

The observer is free to indicate additional elements of the UKPSF which are observed but the identified Dimensions of the UKPSF must be met as a minimum.

Planning and introduction

Please pay especially close attention to the extent to which the observed lesson provided evidence of an awareness of the following Dimensions of the UKPSF

A1 Design and plan learning activities and/or programmes of study (e.g., structure, time management, clear learning outcomes, clear overview of the session, relation to previous session, timely available resources)

A4 Develop effective learning environments and approaches to student support and guidance (e.g., interaction with/amongst students, introduction sets the scene, verbal and nonverbal communication, student reaction, delivery and pace, tone, audibility, rapport, feedback)

Please continue on a separate sheet if required

Teaching and learning

Please pay especially close attention to the extent to which the observed lesson provided evidence of an awareness of the following Dimensions of the UKPSF

K2 Appropriate methods for teaching, learning and assessing in the subject area and at the level of the academic programme * (e.g. teaching methods aligned with learning outcomes, modes of delivery, tutor role, clear and relevant communication of ideas, active/passive methods, summary of main ideas)

K3 How students learn, both generally and within their subject/ disciplinary area(s) (e.g., student engagement and motivation, impact of environment, teaching style, content matches student needs, use of examples)

K4 The use and value of appropriate learning technologies (where appropriate) (e.g., appropriateness, expertise, student engagement, alternatives, cost-effectiveness)

K5 Methods for evaluating the effectiveness of teaching (e.g., seeks student feedback, evaluation of intended learning outcomes, reflection)

V1 Respect individual learners and diverse learning communities (e.g., opportunities for clarifications and questions, active listening, invites questions and facilitates debate, encourages student participation, attends student needs, management of diversity and equality)

V3 Use evidence-informed approaches and the outcomes from research, scholarship and continuing professional development (e.g., rationale for methods, evidence-informed methods and tutor behaviour)

Please continue on a separate sheet if required

Part 3: Post-Observation Evaluation Meeting

Date: ………………….

Observer: ……………………………………………………………………………………

Observee: ……………………………………………………………………………………

Constructive feedback will:

- Allow the observee to describe the class and say how they felt the lesson went before the observer makes any further comment. The observee might reflect on whether the observation process had affected the session in any way, for example, in their behaviour or that of the students.
- Positively state what the observer considers the observee to have done with skill, insight, competence, etc. The observer will provide evidence or instances of any claims made.
- Identify what did not appear to be so successful, or any areas in which some difficulties were observed. It is important that specific instances are cited as evidence for any comment about what did not go so well. Rather provide the opportunity to discuss the issues raised.
- Provide adequate opportunity to discuss any matters of concern with the observee and indicate exactly which Dimensions of the UKPSF were not evidenced.
- Provide an opportunity to identify constructive solutions to any agreed difficulties, including sources of professional development and support.
- Feedback may include suggestions but should not include advice: remember there are lots of ways of being a good teacher.
- Focus on specific issues rather than generalised description or evaluation.
- Link comments explicitly to the UKPSF Dimensions of Practice.

Please make a clear distinction between recommendations for ongoing professional development, and issues that need to be addressed as a matter of urgency.

Strengths to build on:

Agreed areas for possible future development:

Name of observer:

Signature of observer:

Fellowship Status of Observer: FHEA/ SFHEA (delete as appropriate)

Date:

Name of observee:

Signature of observee:

Date:

Part 4: Observee's reflections on the observation process and feedback received

Amongst other aspects, please consider the following:

- How was your experience and what perception do you have of the observation process?
- Are there differences between the observer's and your perception of the teaching session?
- Were you surprised by the observer's comments? Why?
- Amongst the strengths to build on and the positive aspects of the session, which do you think are the most important for your teaching practice? Why?
- How do you plan to take forward the identified areas for further development? What actions could you implement in your next teaching session?

Please continue on a separate sheet if required

TESTIMONIAL FOR TEACHING PRACTICE

Name of Candidate:

Name of Testimonial Provider:

Relationship to Candidate:

Evidence used as basis of testimonial (tick all that apply):

- Line manager
- Annual appraiser of candidate
- Direct observation of teaching activities
- Learner feedback on the candidate's teaching
- Colleague or peer feedback on the candidate's teaching
- Other (please specify):

Please complete all relevant sections of this form.

Brief Job description of the candidate's role in relation to organising, managing or providing educational activity

Please provide examples of at least two specific teaching sessions organised/ delivered by the candidate, focussing on the educator's role within these and feedback received on the sessions

Please highlight any notable strengths identified in relation to their tutor role

Please highlight any areas for improvement identified in relation to their tutor role

Other general comments

Would you recommend this individual for a tutor role in your organisation? Please provide reasons for your answer.

Testimonial Provider's signature:

Designation:

Date:

2.13.4 Professional Development Log Assessment Form for HEA Recognition Status

**Student Name:**	**Category of Fellowship Sought: D1/ D2**
**Assessment of Evidence within PDL, Teaching Observation and Portfolio against the UK Professional Standards Framework (UKPSF) (2011) for D1 (Associate Fellowship) OR D2 (Fellowship) of HEA.**	
**Submission Date:**	

DIMENSIONS OF UKPSF, 2011

**Areas of Activity**	**Appropriate Evidence Present Yes/ No**
A1. Design and plan learning activities and/or programmes of study.	
A2. Teach and/or support learning	
A3. Assess and give feedback to learners.	
A4. Develop effective environments and approaches to student support and guidance.	
A5. Engage in continuing professional development in subjects/disciplines and their pedagogy, incorporating research, scholarship and the evaluation of professional practices.	
**Core knowledge**	**Appropriate Evidence present Yes/No**
K1. The subject material.	
K2. Appropriate methods for teaching, learning and assessing in the subject area and at the level of the academic programme	
K3. How students learn, both generally and within their subject/disciplinary area(s)	
K4. The use and value of appropriate learning technologies	
K5. Methods for evaluating the effectiveness of teaching.	
K6. The implications of quality assurance and quality enhancement for academic and professional practice with a particular focus on teaching.	
**Professional values**	**Appropriate Evidence present Yes/No**
V1. Respect for individual learners and diverse learning communities	
V2. Promote participation in higher education and equality of opportunity for learners	
V3. Use-evidence informed approaches and the outcomes from research, scholarship and continuing professional development	

V4. Acknowledge the wider context in which higher education operates recognising the implications for professional practice	

UKPSF (2011) CRITERIA FOR RECOGNITION STATUS

**Descriptor 1 (D1)**	**Descriptor 2 (D2)**
Demonstrates an understanding of specific aspects of effective teaching, learning support methods and student learning. Individuals should be able to provide evidence of:	Demonstrates a broad understanding of effective approaches to teaching and learning support as key contributions to high quality student learning. Individuals should be able to provide evidence of:
I. Successful engagement with **at least two** of the five Areas of Activity	I. Successful engagement **across all five** Areas of Activity
II. Appropriate Core Knowledge and understanding of **at least K1 and K2**	II. Appropriate knowledge and understanding **across all aspects** of Core Knowledge
III. A commitment to **appropriate** Professional Values in facilitating others' learning	III. A commitment to **all four** Professional Values
IV. Successful engagement in appropriate teaching and practices related to these Areas of Activity	IV. Successful engagement in appropriate teaching practices related to the Areas of Activity
V. Relevant professional practices, subject and pedagogic research and/or scholarship within the above activities	V. Successful incorporation of subject and pedagogic research and/ or scholarship within the above activities, as part of an integrated approach to academic practice
VI. Successful engagement, where appropriate, in professional development activity related to teaching, learning and assessment responsibilities	VI. Successful engagement in continuing professional development in relation to teaching, learning, assessment and, where appropriate, related professional practices

Recognition Decision Reached: D1*/ D2*/ Further information needed (delete as appropriate)
Comments:

Feedback to Student:

Signature of Assessor (Course Team): ……………………………………………………………………………………
Recognition Status of Assessor: FHEA/ SFHEA (delete as appropriate)

Reviewer (External to Course Team): ……………………………………………………………………………………
Recognition Status of Reviewer: FHEA/ SFHEA (delete as appropriate)

If selected for external review through sampling:
Reviewer (External Assessor): ……………………………………………………………………………………………
Recognition Status of Reviewer: PFHEA

Dimensions of the UKPSF

Areas of Activity

- A1 Design and plan learning activities and/or programmes of study
- A2 Teach and/or support learning
- A3 Assess and give feedback to learners
- A4 Develop effective learning environments and approaches to student support and guidance
- A5 Engage in continuing professional development in subjects/disciplines and their pedagogy, incorporating research, scholarship and the evaluation of professional practices

Core Knowledge

- K1 The subject material
- K2 Appropriate methods for teaching, learning and assessing in the subject area and at the level of the academic programme
- K3 How students learn, both generally and within their subject/ disciplinary area(s)
- K4 The use and value of appropriate learning technologies
- K5 Methods for evaluating the effectiveness of teaching
- K6 The implications of quality assurance and quality enhancement for academic and professional practice with a particular focus on teaching

Professional Values

- V1 Respect individual learners and diverse learning communities
- V2 Promote participation in higher education and equality of opportunity for learners
- V3 Use evidence-informed approaches and the outcomes from research, scholarship and continuing professional development
- V4 Acknowledge the wider context in which higher education operates recognising the implications for professional practice

UKPSF 2011

2.13.5 HEA Professional Recognition Judgements & Quality Assurance Process

September – December: students map previous educator experience and collect new evidence for portfolio against UKPSF dimensions at D1/D2; identify any significant gaps in portfolio during module activities, supported by the course team (FHEA/SFHEA); and submit module assessment reflecting on their development as an educator. Students are strongly encouraged to undergo formative teaching observation.

January – July: students develop a learning contract for a work-based educational project with their supervisor (FHEA/SFHEA), carry out and evaluate the project; summative assessment includes verbal presentation and written assignment reflecting on the project and their professional development; update mapping of module activities against UKPSF. Students undergo teaching observation if applying for professional recognition.

July – August: students wishing to apply for HEA professional recognition complete and submit a professional development log (PDL) and action plan with reflective commentary in relation to UKPSF dimensions at D2/D1, a portfolio of evidence including a teaching observation or testimonials. Two SFHEAs from the MClinEd course team will assesses each PDL, portfolio, etc and makes a professional recognition judgement. (D2/D1).

September-October: A trained assessor (SFHEA), external to the PGCertClinEd course team, will review recognition decisions against PDLs/ portfolios for both PGCert-ClinEd and PGCert-HEP for consistency across courses.

A sample of 10% of the successful PDLs and all unsuccessful PDLs are sent to the external assessor (PFHEA) or final checking and confirmation of recognition status.

External assessor, who is a PFHEA, receives and reviews the sample in October and writes their report.

A document summarising professional recognition decisions made that year is submitted to the Faculty postgraduate taught programmes exam board (November) for information & recording.

November –December: Students are notified by course director of outcome (AFHEA/ FHEA) or given further support to develop and re-submit their application for Fellowship.

November-December: Additional information required is obtained from successful individuals and names are uploaded to the HEA database by the Course Director.

Summary of professional recognition decisions made each year, including results of QA checks carried out by external reviewers, is forwarded to the Faculty Learning & Teaching Quality Committee for QA purposes.

2.14 Professional Recognition (Membership of the Academy of Medical Educators)
The PGCertClinEd modules/course (standard, online and primary care modes) have been accredited by the Academy of Medical Educators (AoME) for professional recognition at membership level. This means that all individuals completing the PGCertClinEd modules/course successfully will be eligible to apply for full membership of the Academy of Medical Educators without the need to go through the full application and assessment process.

You will be required to complete the 500 word reflective statement and provide details of a supporter (e.g. an existing Member or Fellow of the Academy, or your Course Director) who can provide us with further information, if required. You will not need to complete the CPD and peer review sections (upon uploading your course certificate, the application form will automatically skip this section).

The AoME membership application form is available here. Membership of the Academy allows the use of the post-nominal letters MAcadMEd, and signifies that the individual has been judged to meet the Academy's Professional Standards for Medical Educators at an appropriate level.

APPENDICES

APPENDICES RE UNDERTAKING AND SUBMITTING ASSIGNMENTS

I. Assessment Guidelines
II. Marking of Student Work

APPENDIX I: ASSESSMENT GUIDELINES

PRIOR TO SUBMITTING YOUR ASSIGNMENT

Prior to submitting your assignment, you should make sure that it conforms to the assignment guidelines detailed in this Appendix, this will help you to avoid penalties. Please refer to the University Student Handbook (Taught Programmes) for guidance on assessments and submission of coursework.

- **Anonymity and Confidentiality Guidelines** – section 1a - FMH rules and guidance to ensure protection of identity of people and places and to maintain their confidences where appropriate.

- **Academic Writing** – The UEA Learning Enhancement Team within Student Support Services provide a variety of resources on academic writing and study skills, including guidance on planning and writing essays and dissertations, as well as referencing and plagiarism. These guides are available here. For all written work submitted for formative or summative assessments and dissertations, please adhere to the following presentation style:

- Use headings and sub-headings as required
- Use Calibri, Arial or Times New Roman font, minimum 11 pt
- Minimum margin 1.5cm on all sides
- Line spacing 1.5 or double
- Insert page numbers.
- Use the Vancouver or Harvard referencing style

I.a. ANONYMITY AND CONFIDENTIALITY GUIDELINES

General principles and expectations:

During your professional programme, you will often be engaging with and/or writing about information that is confidential in nature. Confidential means that it is *private*, and has been entrusted to you for study purposes on the strict understanding that it will not be divulged to third parties. If any third party is interested in the information, you must verify whether they are entitled to it.

In some instances, you may discuss principles around confidential material or draw upon practice experience in an anonymised form. However, it is easy to unintentionally disclose the source of the materials and thereby breach a confidence. It is therefore essential that you do not disclose more information about an individual or scenario than is essential for your purpose.

Types of information to be protected:

As a general rule, none of the following should be named or otherwise specifically identified without prior formal written consent being obtained from the individual and/or institution, and attached as an appendix to the assignment:

- Patients, clients, service users
- Carers, relatives or significant others
- Members of clinical or educational staff

Consideration should also be exercised in the identification of:

- NHS Trusts

- Hospitals, wards, or departments
- Any other placement areas
- UEA, schools of study or specific student cohorts

Information must not be disclosed where it is unlawful to disclose it by reason of the common law or any legislation, including the Data Protection Act 1998.

This means that inclusion of information in your work, such as names, dates of birth, contact details, clinical locations and photographs, or any other material through which an individual might be identified is prohibited.

It is insufficient to use Tipp-Ex or other such materials to delete confidential information in submitted work since this may not adequately obscure the information from view. If a pseudonym is used to preserve anonymity, this must be made clear to the reader in the main body of the assignment.

Information that can be used:
There are three ways you can legitimately write about a specific individual or institution:

- You can seek the individual's or institution's *written consent*, where such consent will state exactly what information is to be used, and how. A copy of the written consent statement must be submitted as part of your written work.
- You can draw information from the public domain, in which case you should provide a clear and precise reference to the book, journal or website.
- Policies, protocols and guidelines issued by the following NHS Trusts may also be used, with appropriate referencing, without further explicit permission:
  - Norfolk and Norwich University Hospitals Foundation Trust
  - Queen Elizabeth Hospital King's Lynn NHS trust
  - James Paget University Hospitals NHS Foundation Trust
  - Norfolk and Suffolk NHS Foundation Trust
  - Norfolk Community Health and Care NHS Trust.

Best Practice and professional guidance:
Best practice changes over time and is formulated for each profession through specific professional codes of conduct. You should at all times ensure that you are familiar with and follow the code of conduct for your professions.

Examples of these are given below:

- Royal College of Speech & Language Therapists:
  http://www.rcslt.org/speech_and_language_therapy/standards/professional_standards_cq3
- Nursing and Midwifery Council:
  http://www.nmc-uk.org
- College of Occupational Therapists:
  http://www.cot.co.uk/standards-ethics/standards-ethics
- Chartered Society of Physiotherapy:
  http://www.csp.org.uk/thecsp/rulesofconduct/rulesofprofessionalconduct.cfm
- General Medical Council:
  http://www.gmc-uk.org/guidance/ethical_guidance/confidentiality.asp
- British Psychological Society:
  https://www.bps.org.uk/Code of Ethics and Conduct 2018.pdf

Support within FMH:
In our professional programmes, we appreciate that there are situations where the question over how confidentiality should be maintained is not clear-cut. Because of this there will be an opportunity to clarify your understandings with academic staff in the preparation of assignment tasks.

'Assessment-specific' guidance will also be provided by Module Organisers regarding any particular considerations that may apply to atypical coursework or assessment activity (e.g. use of video-work, testimonials etc.)

Actions following the identification of a breach of confidentiality:
A framework is provided below which summarises the considerations and actions that may arise following identification of a breach of confidentiality within students work.

It is underpinned by the shared view across the Schools within the FMH that a failure to protect confidential information is primarily of professional concern.

It is also recognised that it is possible where a breach has occurred that a student may also not meet relevant assessment learning outcomes which refer to themes of professional behaviour/ awareness and therefore may receive a referral or fail grade.

BREACH OF CONFIDENTIALTY FRAMEWORK
This framework will be used where a breach of confidentiality is identified in work submitted for assessment and will inform judgements made to determine the 'level' of that breach. It will then be used to identify the actions indicated in each instance.

Please be aware that the examples given are provided to indicate the type of scenarios that may present but is not an exhaustive list.

Where the level of breach is inconsistent across the differing criteria, an overarching outcome will be identified which appears to most accurately reflect the context in which the breach has occurred.

CRITERIA	LOW LEVEL	MEDIUM LEVEL	HIGH LEVEL
**Academic Experience of the Student**	**An inexperienced student who may be unaware of the expected practice within academic work.**<br><br>*For example:*<br>a first-year student on a programme leading to a professional qualification.<br><br>or within the first written submission of post-qualifying study where there is no other recent	**A student who is likely to be aware of expected practice.**<br><br>*For example:*<br>a student who is beyond the first year of a programme leading to a professional qualification but before the final year.<br><br>a student who holds a professional registration who is beyond their first	**An experienced student who is aware of expected practice.**<br><br>*For example:*<br>a student on a programme leading to a professional qualification in their final year.<br><br>a student who holds a professional registration who has completed more than a year of post-qualifying study.

	relevant study experience.<br><br>a student who presents significant mitigation.<br><br>where there are significant cultural considerations.	module of study but still within the first year.<br><br>any student after completion of known instruction regarding the importance of maintaining confidentiality in coursework.<br><br>a student who has received a previous LOW level warning regarding breach of confidentiality.	a student who has received a previous, MEDIUM or HIGH level warning, sanction or fitness to practice referral relating to a breach of confidence.
**Nature of the Breach of Confidence**	**Raises only minor professional concern.**<br><br>*For example:*<br>appears to be an oversight on the part of the student who has ensured confidence elsewhere in the work.<br><br>includes identification of a non-specific geographical area.<br><br>identification of a large organisation.	**Is a cause of significant concern.**<br><br>*For example*:<br>identification of a specific practice setting.<br><br>inclusion of unnecessary detail that may jeopardise confidentiality of individuals or the care context.<br><br>lack of due regard to the protection of confidence such as inadequate 'blacking out' or removal of confidential information.	**Is a cause for major concern and clearly contravenes the relevant professional code.**<br><br>*For example:*<br>explicit identification of an individual (service user, carer or practitioner).<br><br>inclusion of unnecessary detail that indirectly breaches the confidentiality of an individual.<br><br>apparent disregard for the importance of protection of confidence.
**Extent of the Breach of Confidence**	**Minor.**<br><br>*For example:*<br>in one instance within the body of the work<br><br>in one instance within supplementary materials; appendices etc.<br><br>where the student appears to have taken steps to ensure	**Significant.**<br><br>*For example:*<br>two or three instances within a piece of work.<br><br>on one instance within a piece of work but on more than one occasion such as in multiple portfolio elements.	**Substantial.**<br><br>*For example:*<br>throughout the work.<br><br>in several instances.<br><br>in all sections of the work.

	confidentiality throughout the majority of the work.		
**Expected Level of Professional Awareness**	**The student would not be expected to have awareness of the professional expectations regarding protection of confidentiality.**<br><br>*For example:*<br>a student on a programme leading to professional qualification within the first semester of study without any previous experience in a professional environment.<br><br>a student undertaking pre-qualifying study within the first semester of study who has just left school.<br><br>a student undertaking pre-qualifying study within the first semester of study who has not worked previously in a care context.<br><br>a student who has not received any explicit guidance (in theory or practice) regarding the necessity to protect confidential information.	**The student would be expected to be aware of the need to ensure confidentiality but may not fully appreciate the range of implications arising from this or has superficial understanding.**<br><br>*For example:*<br>a student on a programme leading to a professional qualification within the first semester but who has relevant practice experience.<br><br>a student on a programme leading to a professional qualification who is beyond the first year but not yet in the final year.<br><br>a student who has received a previous LOW level warning regarding breach of confidentiality.	**The student is expected to be fully aware of the necessity to protect confidence.**<br><br>*For example*:<br>any registered practitioner.<br><br>a senior student on a programme leading to a professional qualification.<br><br>a student on a programme leading to a professional qualification who has received a previous, MEDIUM or HIGH level warning, sanction or fitness to practice referral relating to a breach of confidence;

The following action will be taken in relation to the overarching level of breach identified.

In all instances the School’s designated Academic/ LTS lead will be notified in order that a record can be made of the student’s registration no., level of breach identified, and action taken.

OVERALL OUTCOME	LOW LEVEL BREACH	MEDIUM LEVEL BREACH	HIGH LEVEL BREACH
**ACTION TO BE TAKEN**	The nominated School Lead should be informed.<br><br>and<br><br>A written warning should be given on assessment feedback documentation by the marker identifying the specific nature of the breach.<br><br>and<br><br>Where a script has also received a referral grade the breach MUST be rectified on resubmission.	The nominated School Lead should be informed.<br><br>and<br><br>A written warning should be given on assessment feedback documentation by the marker identifying the specific nature of the breach.<br><br>and<br><br>Where a script has also received a referral grade the breach MUST be rectified on resubmission.<br><br>and<br><br>The marker **may** refer the case to the School lead for Fitness to Practice for consideration.	The nominated School Lead should be informed.<br><br>and<br><br>A written warning should be given on assessment feedback documentation by the marker identifying the specific nature of the breach.<br><br>and<br><br>Where a script has also received a referral grade the breach MUST be rectified on resubmission.<br><br>and<br><br>The marker **should** refer the case to the School lead for Fitness to Practice for consideration.

I.b. PLAGIARISM AND COLLUSION

The University takes very seriously cases of plagiarism or collusion. Students who deliberately plagiarise or collude threaten the values and beliefs that underpin academic work and devalue the integrity of the University's awards. In proven cases, offenders shall be punished, and the punishment may extend to failing their degree, temporary suspension or expulsion from further study at the University if the case comes before a Discipline Committee of the University. Plagiarism and collusion, at any stage of a student's course, whether discovered before or after graduation, will be investigated and dealt with appropriately by the University.

Please refer to the University Student Handbook (Taught Programmes) for further guidance regarding plagiarism and collusion.

APPENDIX II: MARKING OF STUDENT WORK

II.a. MARKING/FEEDBACK

The University Senate Scales at Master's Level aid the marking and feedback of assessed Coursework, Dissertations and Oral Presentations. The tables for the Senate Scale can be viewed on the LTS website via this link: https://portal.uea.ac.uk/learning-and-teaching/staff/assessment/marking

Coursework is marked using a rubric that combines specific criteria for each assessment (see module assessment section on Blackboard) and the University senate scale.

Failure to submit an assignment automatically generates a 'Clear Fail', 0%.

The Faculty is committed to providing you with feedback and provisional results on summative assessments as quickly as possible.

For coursework (written assignments), the University policy is for feedback and provisional marks to be returned to students within 20 working days of the published submission date. Where we are unable to meet the norm, you will be notified, and an explanation given.

For dissertations, portfolios, and projects the norm will be 30 working days.

Work that is submitted after the published deadline, including approved extensions, may take longer than the 20-working day timescale but will normally be within 30 days. This is due to missing the allocated time slot for marking based on the original deadline.

II.b. PROVISIONAL MARKS

All marks awarded are **provisional** until they have been ratified by the Board of Examiners. Before your marks are ratified by the Board of Examiners, feedback will be provided to you in the form both of written comments and a provisional mark. Please refer to the UEA Student Handbook (Taught Programmes) https://portal.uea.ac.uk/learning-and-teaching/students/getting-started/handbooks

II.c. THE BOARD OF EXAMINERS CONFIRMATION OF MARKS

The Board of Examiners meets three times a year (in November, March and June).

The Board of Examiners is made up of experienced internal and external examiners who monitor students work, scrutinise the quality of marking processes and critically compare UEA standards of student work and marking to that in other equivalent institutions. During the course of its work, the Board of Examiners has the power to moderate marks and assessment results in light of their findings from the above activities.

Final results and confirmed marks will be issued following the meeting of the Board of Examiners. A mark of 50% or more for any assessed component will be treated as a pass mark and will qualify for credit. A candidate who receives a mark of 49.99% or below will be referred to reassessment.

II.d. EXTENUATING CIRCUMSTANCES

If you have any extenuating circumstances that have impacted on your coursework or other assessments, please complete and submit an extenuating circumstances form to the LTS hub. Further details are

available on the LTS website: https://portal.uea.ac.uk/learning-and-teaching/students/advice-on-difficulties/extenuating-circumstances

II.e. THE BOARD OF EXAMINERS AND REASSESSMENT
In the event of a failed assessment, the Board of Examiners will decide whether to confirm the mark and consider whether or not you can be reassessed. You will then be sent a letter notifying you of the Board of Examiners' decision. If you are eligible to be reassessed, you will be provided with instructions in the letter and you will be charged a reassessment fee. You are allowed **three months from the date of the letter to resubmit your assignment**. You will not be allowed reassessment in any one module, on more than one occasion.

Your module co-ordinator is the person to whom you should speak regarding the nature of the work you are to submit for reassessment, as you will be notified in the letter offering resubmission. Students are advised to read and respond to the marker's feedback when undertaking their reassessment.

II.f. CANDIDATES ARE ADVISED THAT:

1. The deliberations of Boards of Examiners are confidential. Examiners are not authorised to inform candidates of any discussion that may have taken place in the Board's meeting.

2. Examiners are required to make academic judgements about candidates' performance and marks are a guide to examiners in making those judgements. However, other factors may be taken into account and candidates should be aware that a particular number or pattern of marks does not necessarily lead to a given result.

II.g. APPEALS, COMPLAINTS, AND MATTERS OF CONCERN
All appeals, complaints and matters of concern will be investigated in a timely and fair manner and in line with UEA policies and procedures.

If, as a student, you believe that you have grounds for an academic appeal or have a complaint in relation to decision relating to your academic work you should, in the first instance, discuss this with the Course Leader. If you do not feel that your concerns have been appropriately dealt with then you should pursue your complaint via the UEA Appeals and Complaints procedure which can be accessed via this link: https://portal.uea.ac.uk/documents/6207125/7465906/Section+3+Academic+Appeals+and+Complaints+Procedure.pdf

If an individual makes a complaint to the HEA regarding their experience on an accredited programme, the HEA will direct the complainant to the appeals and/or complaints procedure at the accredited institution wherever an issue raised relates to institutional policy/procedure or support provided. The HEA will not intervene in matters between an institution and participant relating to institutional policy or procedure but reserves the right to intervene in instances where a complaint/appeal might relate to HEA standards or policy requirements or deviation from an accredited process. Should a complaint relate to a potential compromise of HEA accredited process, policy requirements or standards, the HEA will investigate in line with the HEA Complaints Policy which can be accessed at this link: https://www.heacademy.ac.uk/system/files/downloads/compliments_and_complaints.pdf

The HEA Code of Practice, which can be accessed here: https://www.heacademy.ac.uk/system/files/downloads/Code_Of_Practice_0.pdf sets out principles and expectations for HEA Fellows and the HEA reserves the right to remove an individual's HEA Fellowship on the grounds of academic or professional misconduct following formal investigation by the HEA and associated HEA disciplinary procedures.

APPENDIX III: LEARNING CONTRACT FOR WORK-BASED PROJECT

**Establish a relevant work-based learning need/ goal relating to an aspect of leadership in teaching, learning and assessment.**	
**Specify SMART objectives within the goal.**	
**Specify learning strategies and resources to achieve the objectives.**	
**Specify what is to be assessed.**	
**Specify how evidence of accomplishment will be measured.**	

Name of Student:

Signature of Student:

Name of Project Supervisor:

Signature of Project Supervisor: **Date:**

STUDENT NOTES

STUDENT NOTES

STUDENT NOTES

STUDENT NOTES

STUDENT NOTES

STUDENT NOTES

STUDENT NOTES

STUDENT NOTES
