# Supplementary material for: Implementation and lessons learned from 2 online interprofessional faculty development programs for improving educational practice in the health professions in Chile and the United Kingdom from 2018 to 2021
Source: J Educ Eval Health Prof. 2021 Aug 9;18:21. doi: 10.3352/jeehp.2021.18.21 (PMC8441097; doi:10.3352/jeehp.2021.18.21)
Supplement: Supplementary file 2 — Supplement 2. Postgraduate diploma in health professions education course program/handbook. [file jeehp-18-21-suppl2.pdf]

DIPLOMADO EN DOCENCIA UNIVERSITARIA EN CIENCIAS DE LA SALUD
2020-2021

Universidad de los Andes
Facultad de Odontología

MISION DE LA UNIVERSIDAD

La Universidad de los Andes es una institución que busca profundizar en el conocimiento de todos los ámbitos del saber y contribuir a la formación integral de sus estudiantes. Quiere promover el diálogo académico, el trabajo bien hecho y el afán de servicio a la sociedad. Anhela irradiar un modo de vida coherente con las enseñanzas de la Iglesia católica.

MISION DE LA FACULTAD

Servir a la sociedad formando profesionales de la salud bucal íntegros, competentes, con vocación de servicio y trabajo en equipo de salud, comprometidos con el cuidado de la salud general del paciente, mediante labores de vanguardia e innovación, en las áreas de docencia, investigación, formación continua y vinculación con la comunidad, las cuales se desarrollan en el marco del ideario institucional.

IDENTIFICACIÓN DEL PROGRAMA

**Programa**	Diplomado en Docencia Universitaria en Ciencias de la Salud		
**Unidad Académica**	Facultad de Odontología		
**Código**	DDUC		
**Créditos SCT-Chile**	12		
**Tipo de Programa**	Online		
**Duración**	2 semestres		
**Horas de dedicación**	Totales: 360	Horas Sincrónicas: 10	Horas Asincrónicas: 350
**Horario**	• **Fecha de inicio:** Lunes 10 de Agosto, 2020.<br>• **Fecha de término:** Domingo 11 de Julio, 2021<br>• **Actividades sincrónicas grupales online**: 10 horas.<br>**Sesiones Webinars**: 3 sesiones de máximo 2 horas de duración.<br>- Martes 6 de Octubre 2020<br>- Martes 30 de Marzo 2021<br>- Martes 29 de Junio 2021<br>**Sesiones de Preguntas y Respuestas:** 4 sesiones de máximo 1 hora de duración por Módulo, de participación optativa.<br>- Martes 11 de Agosto 2020 (Webinar de bienvenida)<br>- Martes 8 de Diciembre 2020<br>- Martes 6 de Abril 2021<br>- Martes 18 de Mayo 2021<br>- Martes 06 de Julio 2021<br>• **Actividades asincrónicas grupales e individuales online:** 7.5 horas semanales (videos interactivos, lecturas dirigidas, participación en foros, autoestudio, evaluaciones, tutoriales personalizados).<br>• **Periodo de vacaciones de Fiestas Patrias:**<br>- Lunes 14 a Domingo 20 de Septiembre de 2020.<br>• **Periodo de vacaciones de Fin de Año:**<br>- Lunes 21 de Diciembre de 2020 – Domingo 3 de Enero 2021.<br>• **Periodo de vacaciones de Fin de Año:**<br>- Lunes 25 de Enero 2021 – Domingo 28 de Febrero 2021.		
**Requisitos**	1. Dominio del idioma inglés técnico de nivel intermedio/superior con capacidad de comprensión lectora.<br>2. Estar desempeñándose como docente en algún nivel de la Educación Superior al momento de iniciar el programa.<br>3. Manejo computacional nivel usuario. Acceso a internet de banda ancha, cámara y micrófono para sesiones online.		

**Equipo Docente**	Director: Dr. Jorge Tricio Pesce (jtricio@uandes.cl)<br>Coordinador: Dr. César Orsini (corsini@miuandes.cl)<br>Docentes colaboradores e invitados nacionales y extranjeros.

DESCRIPCIÓN DEL PROGRAMA

**Descripción**	El Diplomado en Docencia Universitaria en Ciencias de la Salud es un programa de perfeccionamiento profesional en docencia universitaria de dos semestres de duración, online, y diseñado específicamente para profesionales de la salud que ejercen labores docentes en la Educación Superior.<br><br>El programa comprende los módulos de (1) Aprendizaje-Enseñanza, (2) Evaluación, (3) Currículo, y (4) Liderazgo y Gestión, los cuales son cardinales para desarrollar una práctica docente profesionalizada y transversal a todas las áreas de la salud del quehacer universitario.
**Intención Formativa**	Desarrollar y fortalecer las competencias docentes de los académicos de pregrado y postgrado del área de la salud con el fin de mejorar la calidad de la educación que se entrega a los estudiantes a través de un enfoque profesionalizante y basado en la evidencia de la docencia que transmiten.
**Gran pregunta que guía el programa**	¿Cómo se caracteriza, cuáles son los componentes, y cómo se aplica una docencia de calidad en la Educación en Ciencias de la Salud, considerando sus dimensiones de aprendizaje-enseñanza, evaluación, currículo, y liderazgo y gestión?
**Perfil de Egreso**	**Descripción:** El graduado del programa de Diplomado en Docencia Universitaria en Ciencias de la Salud de la Universidad de los Andes es un profesional que se distingue por ejercer su labor docente en las ciencias de la salud de forma respetuosa, ética y centrada en el alumno. Demuestra conocimientos pedagógicos actualizados en enseñanza y aprendizaje activo, herramientas de evaluación y tutoría, diseño y desarrollo curricular, así como también en liderazgo y gestión, que le permiten mejorar su práctica de tal forma de entregar una educación de calidad e influir positivamente en el aprendizaje de sus alumnos.<br><br>**Áreas de desempeño:** El titulado del programa podrá desempeñarse en la educación superior en salud, tanto a nivel universitario como técnico-profesional.

	**Síntesis de resultados de aprendizaje:**<br><br>**Analizar** los diferentes procesos de aprendizaje **y planificar** estrategias didácticas que favorezcan el aprendizaje de acuerdo con el objetivo pedagógico.<br><br>**Analizar** los principios modernos de evaluación **y planificar** estrategias que guíen, faciliten, y midan el aprendizaje en las profesiones de la salud.<br><br>**Analizar** programas de estudios y syllabus considerando las bases del diseño curricular en retrospectiva.<br><br>**Evaluar** el liderazgo y gestión de su docencia con el objetivo de contribuir al aseguramiento de la calidad del proceso educativo en su aula.
**Requisitos de Aprobación**	El programa se aprueba con un 70% del cumplimiento de cada Resultado de Aprendizaje de forma independiente. La nota final de aprobación del diplomado será el promedio de cada uno de estos Resultados de Aprendizaje. El 100% de la calificación se calcula considerando:<br><br>• Módulo 1: 32%<br>- Ensayo final de módulo 16%<br>- Presentación grupal (webinar) 6%<br>- Autoevaluación: Observación de la Enseñanza 10%<br><br>• Módulo 2: 24%<br>- Ensayo final de módulo 12%<br>- Presentación grupal (webinar) 6%<br>- Autoevaluación: Revisión y mejora de pruebas de selección múltiple 6%<br><br>• Módulo 3: 20%<br>- Ensayo final de módulo 12%<br>- Autoevaluación: Protocolo de Revisión de Programa y Syllabus 8%<br><br>• Módulo 4: 24%<br>- Ensayo final de módulo 12%<br>- Presentación grupal (webinar) 6%<br>- Autoevaluación: Guía para el Reporte de Evaluación de Asignaturas 6%

MÓDULO 1: APRENDIZAJE-ENSEÑANZA

**Resultado de Aprendizaje 1**	**Analizar** los diferentes procesos de aprendizaje **y planificar** estrategias didácticas que favorezcan el aprendizaje de acuerdo con el objetivo pedagógico.
**Descripción del Resultado de Aprendizaje 1**	El logro de este resultado de aprendizaje implica que el docente será capaz de describir y contrastar las diferentes formas a través de las cuales se produce el aprendizaje en sus alumnos, aplicándolas en el ámbito de su docencia actual para facilitar y potenciar el aprendizaje en diferentes situaciones en las ciencias de la salud.<br><br>Además, este resultado de aprendizaje implica que el docente será capaz de analizar y aplicar la evidencia de las estrategias didácticas en las ciencias de la salud, para favorecer un aprendizaje profundo y significativo de sus alumnos en diferentes situaciones.
**Contenidos**	1. Introducción y particularidades de la educación en las ciencias de la salud.<br>2. Aplicación de las teorías del aprendizaje.<br>3. Herramientas para aprender mejor: Neurociencias del aprendizaje.<br>4. ¿Cómo fomentar la motivación óptima y la autodeterminación en los estudiantes?<br>5. Estrategias de enseñanza activas en grupos grandes.<br>6. Estrategias de enseñanza activas en grupos pequeños.<br>7. Diseño de ambientes simulados como recurso de enseñanza.<br>8. Enseñanza y desarrollo del profesionalismo.<br>9. Educación Interprofesional: aprendiendo con, desde y sobre otras disciplinas.<br>10. Habilidades comunicacionales verbales y no verbales del docente.
**Estrategias de aprendizaje-enseñanza**	- Presentaciones online.<br>- Foros interactivos.<br>- Lecturas dirigidas.<br>- Portafolio reflexivo.<br>- Seminarios online con presentaciones grupales (webinars).<br>- Tutoriales uno a uno.<br>- Sesiones de preguntas y respuestas.
**Estrategias de evaluación de los aprendizajes**	**Formativas**<br><br>- Cuestionarios cortos (quiz), en diferentes formatos, en las sesiones online con respuesta y retroalimentación inmediata.<br><br>- <u>Participación en Portafolio Reflexivo</u>: De carácter personal, cada participante desarrollará una reflexión de un máximo de 250 palabras, registrado en un e-portafolio en las unidades que corresponda. La

	reflexión será a partir de una(s) pregunta planteada en relación a la importancia e implicancias del contenido tratado para la Educación en Ciencias de la Salud. Esta participación corresponde a un requisito habilitante y no a una calificación.<br><br>- Participación en foros interactivos: Ciertos contenidos a tratar contarán con foros, en los cuales se plantearán temas y preguntas para estimular el debate. El número de participaciones mediante entradas nuevas y respuestas comentando la perspectiva de sus pares es de carácter ilimitado; no obstante, se requerirá como base una entrada nueva y dos respuestas a un par. Esta participación corresponde a un requisito habilitante y no a una calificación.<br><br>**Sumativas**<br><br>- Presentación grupal (webinar): El curso se dividirá en grupos. Se planteará una pregunta por grupo a través del foro interactivo en una Unidad específica. Se espera que los integrantes de cada grupo se organicen y debatan la respuesta a la pregunta planteada, para luego presentar sus conclusiones en la sesión de webinar correspondiente. Cada grupo contará con 15 minutos para realizar su presentación oral, seguido por 5 minutos de preguntas. El contenido de la presentación será evaluado por los docentes del Programa según una rúbrica (Anexo I).<br><br>- Ensayo final de módulo: Reflexión escrita de aproximadamente 1000 palabras analizando su práctica actual en relación a los contenidos tratados en este resultado de aprendizaje y cómo transferirlos a su práctica pedagógica. Éste será evaluado con una rúbrica (Anexo II) por un docente del Programa.<br><br>- Observación de la enseñanza: Cada participante deberá realizar una grabación de audio y video de su docencia, ya sea en grupos grandes o pequeños, de una duración entre 15 a 45 minutos que abarque el comienzo, desarrollo y término de ésta. Posteriormente, la sesión pregrabada será autoevaluada de acuerdo a una Rúbrica de Autoevaluación (Anexo III), seguida de una reflexión final. El proceso completo será evaluado por un docente del Programa considerando el cumplimiento de los plazos de entrega y etapas correspondientes.
**Recursos de aprendizaje**	La bibliografía básica y complementaria será facilitada por los docentes de cada módulo y corresponderá a artículos provenientes de revistas especializadas en la literatura en Educación en Ciencias de la Salud.

MÓDULO 2: EVALUACIÓN

**Resultado de Aprendizaje 2**	**Analizar** los principios modernos de evaluación y **planificar** estrategias que guíen, faciliten, y midan el aprendizaje en las profesiones de la salud.
**Descripción del Resultado de Aprendizaje 2**	El logro de este resultado de aprendizaje implica que el docente será capaz de analizar componentes fundamentales de la evaluación, así como diseñar herramientas para guiar, facilitar y medir el conocimiento y su aplicación, además de competencias clínicas en ambientes simulados y reales.
**Contenidos**	1. Fundamentos de la evaluación.<br>2. Propósito y utilidad de una evaluación sumativa y formativa.<br>3. Diseño y construcción de una evaluación.<br>4. Herramientas centrales de evaluación: Selección múltiple, desarrollo corto y extendido, OSCE y desempeño clínico.<br>5. Interpretación de resultados de una evaluación.
**Estrategias de aprendizaje- enseñanza**	- Presentaciones online.<br>- Foros interactivos.<br>- Lecturas dirigidas.<br>- Portafolio reflexivo.<br>- Seminarios online con presentaciones grupales (webinars).<br>- Tutoriales uno a uno.<br>- Sesiones de preguntas y respuestas.
**Estrategias de evaluación de los aprendizajes**	**Formativas**<br>- Cuestionarios cortos (quiz), en diferentes formatos, en las sesiones online con respuesta y retroalimentación inmediata.<br>- Participación en Portafolio Reflexivo: De carácter personal, cada participante desarrollará una reflexión de un máximo de 250 palabras, registrado en un e-portafolio en las unidades que corresponda. La reflexión será a partir de una(s) pregunta planteada en relación a la importancia e implicancias del contenido tratado para la Educación en Ciencias de la Salud. Esta participación corresponde a un requisito habilitante y no a una calificación.<br>- Participación en foros interactivos: Ciertos contenidos a tratar contarán con foros, en los cuales se plantearán temas y preguntas para estimular el debate. El número de participaciones mediante entradas nuevas y respuestas comentando la perspectiva de sus pares es de carácter ilimitado; no obstante, se requerirá como base una entrada nueva y dos respuestas a un par. Esta participación corresponde a un requisito habilitante y no a una calificación.

	**Sumativas**<br><br>- Presentación grupal (webinar): El curso se dividirá en grupos. Se planteará una pregunta por grupo a través del foro interactivo en una Unidad específica. Se espera que los integrantes de cada grupo se organicen y debatan la respuesta a la pregunta planteada, para luego presentar sus conclusiones en la sesión de webinar correspondiente. Cada grupo contará con 15 minutos para realizar su presentación oral, seguido por 5 minutos de preguntas. El contenido de la presentación será evaluado por los docentes del Programa según una rúbrica (Anexo I).<br><br>- Ensayo final de módulo: Reflexión escrita de aproximadamente 1000 palabras analizando su práctica actual en relación a los contenidos tratados en este resultado de aprendizaje y cómo transferirlos a su práctica pedagógica. Éste será evaluado con una rúbrica (Anexo II) por un docente del Programa.<br><br>- Revisión de la calidad de evaluaciones de selección múltiple: Cada participante proporcionará una prueba de selección múltiple de la asignatura en la cual realiza docencia (mínimo 30 preguntas). Utilizando un protocolo de Revisión y Mejora de Evaluaciones de Selección Múltiple RME (Anexo IV), cada prueba proporcionada deberá ser autoevaluada considerando todos los ítems de la RME, además de realizar comentarios directos en cada pregunta de la evaluación. Luego, el docente deberá realizar una reflexión. El proceso completo será evaluado por un docente del Programa considerando el cumplimiento de los plazos de entrega y etapas correspondientes.
**Recursos de aprendizaje**	La bibliografía básica y complementaria será facilitada por los docentes de cada módulo y corresponderá a artículos provenientes de revistas especializadas en la literatura en Educación en Ciencias de la Salud.

MÓDULO 3: CURRÍCULO

**Resultado de Aprendizaje 3**	**Analizar** programas de estudios y syllabus considerando las bases del diseño curricular en retrospectiva.
**Descripción del Resultado de Aprendizaje 3**	El logro de este resultado de aprendizaje implica que el docente será capaz de planificar y llevar a cabo la elaboración de un programa de estudio y su calendarización (syllabus), guiado por el alineamiento entre el perfil de egreso, resultados de aprendizaje, evaluación, actividades de aprendizaje, y la carga académica.
**Contenidos**	1. Principios y tipos de currículo.<br>2. Currículos Innovados.<br>3. Diseño de programa y syllabus.
**Estrategias de aprendizaje- enseñanza**	- Presentaciones online.<br>- Foros interactivos.<br>- Lecturas dirigidas.<br>- Portafolio reflexivo.<br>- Tutoriales uno a uno.<br>- Sesiones de preguntas y respuestas.
**Estrategias de evaluación de los aprendizajes**	**Formativas**<br>- Cuestionarios cortos (quiz), en diferentes formatos, en las sesiones online con respuesta y retroalimentación inmediata.<br>- Participación en Portafolio Reflexivo: De carácter personal, cada participante desarrollará una reflexión de un máximo de 250 palabras, registrado en un e-portafolio en las unidades que corresponda. La reflexión será a partir de una(s) pregunta planteada en relación a la importancia e implicancias del contenido tratado para la Educación en Ciencias de la Salud. Esta participación corresponde a un requisito habilitante y no a una calificación.<br>- Participación en foros interactivos: Ciertos contenidos a tratar contarán con foros, en los cuales se plantearán temas y preguntas para estimular el debate. El número de participaciones mediante entradas nuevas y respuestas comentando la perspectiva de sus pares es de carácter ilimitado; no obstante, se requerirá como base una entrada nueva y dos respuestas a un par. Esta participación corresponde a un requisito habilitante y no a una calificación.

	**Sumativas**<br><br>- Ensayo final de módulo: Reflexión escrita de aproximadamente 1000 palabras analizando su práctica actual en relación a los contenidos tratados en este resultado de aprendizaje y cómo transferirlos a su práctica pedagógica. Éste será evaluado con una rúbrica (Anexo II) por un docente del Programa.<br><br>- Revisión de programa y syllabus: Cada participante proporcionará un programa (y syllabus en caso de disponer) de la asignatura en la cual realiza docencia. Utilizando un protocolo de Revisión de Programa y Syllabus (RPS) (anexo V), cada programa proporcionado deberá ser autoevaluado considerando todos los ítems del RPS. Luego, el docente deberá realizar una reflexión. El proceso completo será evaluado por un docente del Programa considerando el cumplimiento de los plazos de entrega y etapas correspondientes.
**Recursos de aprendizaje**	La bibliografía básica y complementaria será facilitada por los docentes de cada módulo y corresponderá a artículos provenientes de revistas especializadas en la literatura en Educación en Ciencias de la Salud.

MÓDULO 4: LIDERAZGO Y GESTIÓN EN EL AULA

**Resultado de Aprendizaje 4**	**Evaluar** el liderazgo y gestión de su docencia con el objetivo de contribuir al aseguramiento de la calidad del proceso educativo en su aula.
**Descripción del Resultado de Aprendizaje 4**	El logro de este resultado de aprendizaje implica que el docente será capaz de evaluar los componentes que favorezcan un liderazgo y gestión efectivos, y desarrollar una propuesta que contribuya al aseguramiento de la calidad de su docencia.
**Contenidos**	1. El liderazgo y gestión educacional como herramientas para procesos de cambios, innovación, y aseguramiento de la calidad.<br>2. Práctica docente basada en la evidencia: ¿dónde, cómo, y por qué indagar?<br>3. Evaluación de asignaturas: Efectividad del proceso de aprendizaje-enseñanza.
**Estrategias de aprendizaje- enseñanza**	- Sesiones interactivas en grupos pequeños y grandes.<br>- Presentaciones online.<br>- Foros interactivos.<br>- Lecturas dirigidas.<br>- Portafolio reflexivo.<br>- Seminarios online con presentaciones grupales (webinars).<br>- Tutoriales uno a uno.<br>- Sesiones de preguntas y respuestas.
**Estrategias de evaluación de los aprendizajes**	**Formativas**<br>- <u>Cuestionarios cortos</u> (quiz), en diferentes formatos, en las sesiones online con respuesta y retroalimentación inmediata.<br>- <u>Participación en Portafolio Reflexivo</u>: De carácter personal, cada participante desarrollará una reflexión de un máximo de 250 palabras, registrado en un e-portafolio en las unidades que corresponda. La reflexión será a partir de una(s) pregunta planteada en relación a la importancia e implicancias del contenido tratado para la Educación en Ciencias de la Salud. Esta participación corresponde a un requisito habilitante y no a una calificación.<br>- <u>Participación en foros interactivos</u>: Ciertos contenidos a tratar contarán con foros, en los cuales se plantearán temas y preguntas para estimular el debate. El número de participaciones mediante entradas nuevas y respuestas comentando la perspectiva de sus pares es de carácter ilimitado; no obstante, se requerirá como base una entrada nueva y dos respuestas a un par. Esta participación corresponde a un requisito habilitante y no a una calificación.

	**Sumativas**<br><br>- <u>Presentación grupal (webinar)</u>: El curso se dividirá en grupos. Se planteará una pregunta por grupo a través del foro interactivo en una Unidad específica. Se espera que los integrantes de cada grupo se organicen y debatan la respuesta a la pregunta planteada, para luego presentar sus conclusiones en la sesión de webinar correspondiente. Cada grupo contará con 15 minutos para realizar su presentación oral, seguido por 5 minutos de preguntas. El contenido de la presentación será evaluado por los docentes del Programa según una rúbrica (Anexo I).<br><br>- <u>Ensayo final de módulo</u>: Reflexión escrita de aproximadamente 1000 palabras analizando su práctica actual en relación a los contenidos tratados en este resultado de aprendizaje y cómo transferirlos a su práctica pedagógica. Éste será evaluado con una rúbrica (Anexo II) por un docente del Programa.<br><br>- <u>Evaluación y propuesta de mejora de asignaturas</u>: Cada participante reportará la evaluación (métodos y resultados) del efecto académico en sus estudiantes de alguna de sus asignaturas ya finalizadas. Basado en esta evaluación, el docente realizará una propuesta para mejorar el efecto académico de dicha asignatura. Utilizando una guía para el Reporte de Evaluación de Asignaturas REA (Anexo VI), cada evaluación y propuesta será autoevaluada seguida por una reflexión. El proceso completo será evaluado por un docente del Programa considerando el cumplimiento de los plazos de entrega y etapas correspondientes.
**Recursos de aprendizaje**	La bibliografía básica y complementaria será facilitada por los docentes de cada módulo y corresponderá a artículos provenientes de revistas especializadas en la literatura en Educación en Ciencias de la Salud.

Universidad de
los Andes

DIPLOMADO EN DOCENCIA UNIVERSITARIA
EN CIENCIAS DE LA SALUD

MÓDULO 1: APRENDIZAJE-ENSEÑANZA

RA 1. **Analizar** los diferentes procesos de aprendizaje **y planificar** estrategias didácticas que favorezcan el aprendizaje de acuerdo al objetivo pedagógico**.**

MÓDULO 2: EVALUACIÓN

RA 2. **Analizar** los principios modernos de evaluación y **planificar** estrategias que guíen, faciliten, y midan el aprendizaje en las profesiones de la salud.

MÓDULO 3: CURRÍCULO

RA 3. **Analizar** programas de estudios y syllabus considerando las bases del diseño curricular en retrospectiva.

MÓDULO 4: LIDERAZGO Y GESTIÓN EN EL AULA

RA 4. **Evaluar** el liderazgo y gestión de su docencia con el objetivo de contribuir al aseguramiento de la calidad del proceso educativo en su aula.

DIPLOMADO EN DOCENCIA UNIVERSITARIA EN CIENCIAS DE LA SALUD
2020-2021
UNIVERSIDAD DE LOS ANDES

Calendario de Actividades Académicas 2020-2021

DIPLOMADO EN DOCENCIA UNIVERSITARIA EN CIENCIAS DE LA SALUD			
**Unidad**	**FECHA**	**Módulo**	**Contenidos a tratar**
	10-16 Ago		Bienvenida y orientación al Diplomado<br>**Webinar de bienvenida**
1	17-23 Ago		Introducción y particularidades de la Educación en las Ciencias de la Salud
2	24-30 Ago		Aplicación de las teorías del aprendizaje
	31 Ago-6 Sept		
	7-13 Sep		
	14-20 Sep		Semana de Fiestas Patrias
3	21-27 Sep		Herramientas para aprender mejor: Neurociencias del aprendizaje
4	28 Sep- 4 Oct		¿Cómo fomentar la motivación óptima y la autodeterminación en los estudiantes?
5	5-11 Oct		**Semana de Webinar Módulo 1**
6	12-18 Oct		Estrategias de enseñanza activas en grupos grandes
	19-25 Oct		
7	26 Oct-1 Nov		Estrategias de enseñanza activas en grupos pequeños
	2- 8 Nov		

8	9-15 Nov		Diseño de ambientes simulados como recurso de enseñanza
9	16-22 Nov		Enseñanza y desarrollo del profesionalismo
10	23-29 Nov		Educación Interprofesional: aprendiendo con, desde y sobre otras disciplinas
11	30 Nov-6 Dic		Habilidades comunicacionales verbales y no verbales del docente
12	7-13 Dic		**Semana de confección y desarrollo de Ensayo Módulo 1**<br>**Sesión online de Preguntas y Respuestas Módulo 1**
13	14-20 Dic		Fundamentos de la Evaluación
	21-27 Dic	Navidad y Año Nuevo	
	28 Dic-3 Ene		
14	4-10 Ene		Propósito y utilidad de una evaluación sumativa y formativa
	11-17 Ene		
15	18- 24 Ene		Diseño y construcción de una evaluación
	25 Ene- 28 Feb	Receso Universitario	
16	1-7 Mar		Herramientas centrales de evaluación: Selección múltiple, desarrollo corto y extendido, OSCE y desempeño clínico
	8-14 Mar		
	15-21 Mar		
17	22-28 Mar		Interpretación de resultados de una evaluación

18	29 Mar-4 Abr		Semana de **Webinar Módulo 2**
19	5-11 Abr		**Semana de confección y desarrollo de Ensayo Módulo 2**<br>**Sesión online de Preguntas y Respuestas Módulo 2**
20	12-18 Abr		Principios y tipos de currículo
21	19-25 Abr		Currículos Innovados
22	26 Abr-2 May		Diseño de programa y syllabus
	3-9 May		
	10-16 May		
23	17-23 May		**Semana de confección y desarrollo de Ensayo Módulo 3**<br>**Sesión online de Preguntas y Respuestas Módulo 3**
24	24-30 May		El liderazgo y gestión educacional como herramientas para procesos de cambios, innovación, y aseguramiento de la calidad
25	31 May-6 Jun		Práctica docente basada en la evidencia: ¿dónde, cómo, y por qué indagar?
26	7-13 Jun		Evaluación de asignaturas: Efectividad del proceso de aprendizaje-enseñanza
	14-20 Jun		
	21-27 Jun		
27	28 Jun-4 Jul		**Semana Webinar Módulo 4**
28	5-11 Jul		**Semana de confección y desarrollo de Ensayo Módulo 4**<br>**Sesión online de Preguntas y Respuestas Módulo 4**
